# Deciphering Structural Determinants Distinguishing Active from Inactive Cell-Penetrating Peptides for Cytosolic mRNA Delivery

**DOI:** 10.1021/acs.bioconjchem.3c00346

**Published:** 2023-09-21

**Authors:** Rik Oude Egberink, Alexander H. van Asbeck, Milou Boswinkel, Grigor Muradjan, Jürgen Dieker, Roland Brock

**Affiliations:** † Department of Medical BioSciences, Research Institute for Medical Innovation, Radboud University Medical Center, 6525 GA Nijmegen, The Netherlands; ‡ Department of Medical Biochemistry, College of Medicine and Medical Sciences, Arabian Gulf University, Manama 329, Bahrain

## Abstract

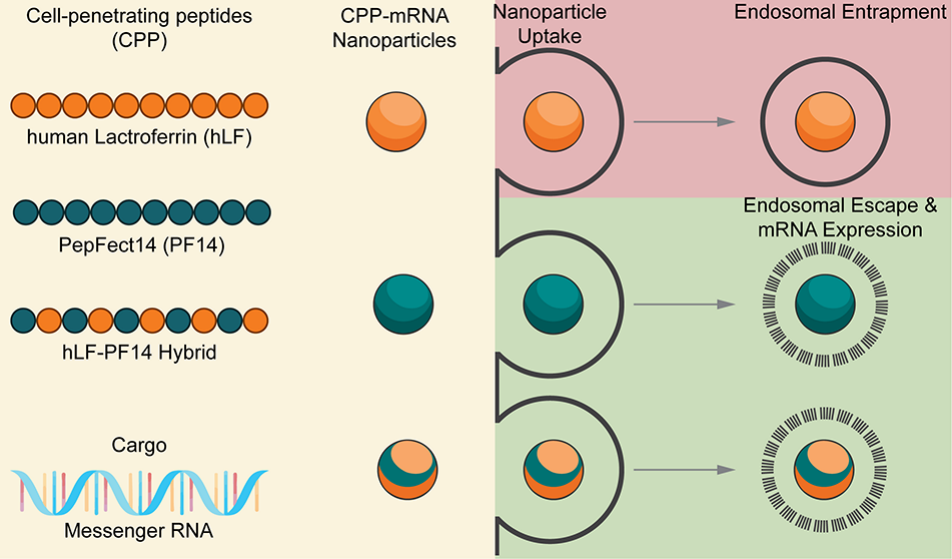

The formation of noncovalent complexes by mixing of positively charged polymers with negatively charged oligonucleotides (ONs) is a widely explored concept in nanomedicine to achieve cellular delivery of ONs. Uptake of ON complexes occurs through endocytosis, which then requires release of ON from endosomes. As one type of polymer, cell-penetrating peptides (CPPs) are being used which are peptides of about 8–30 amino acids in length. However, only a few CPPs yield effective cytosolic ON delivery and activity. Several strategies have been devised to increase cellular uptake and enhance endosomal release, among which an increase of osmotic pressure through the so-called proton sponge effect, disruption of membrane integrity through membrane activity, and disulfide-mediated polymerization. Here, we address the relevance of these concepts for mRNA delivery by incorporating structural features into the human lactoferrin-derived CPP, which shows uptake but not delivery. The incorporation of histidines was explored to address osmotic pressure and structural motifs of the delivery-active CPP PepFect14 (PF14) to address membrane disturbance, and finally, the impact of polymerization was explored. Whereas oligomerization increased the stability of polyplexes against heparin-induced decomplexation, neither this approach nor the incorporation of histidine residues to promote a proton-sponge effect yielded activity. Also, the replacement of arginine residues with lysine or ornithine residues, as in PF14, was without effect, even though all polyplexes showed cellular uptake. Ultimately, sufficient activity could only be achieved by transferring amphipathic sequence motifs from PF14 into the hLF context with some benefit of oligomerization demonstrating overarching principles of delivery for CPPs, lipid nanoparticles, and other types of delivery polymers.

## Introduction

Cell-penetrating peptides (CPPs) are widely explored as vehicles to enhance the cellular delivery of associated cargos that do not enter cells unaided.^1,2^ Ideally, uptake occurs by direct permeation through the plasma membrane. However, this uptake mechanism has only been observed for amphipathic peptides with membrane activity,^3,4^ and for arginine-rich peptides either at high concentration^5^ or upon the incorporation of additional conformational constraints,^6,7^ change of lipid composition of the plasma membrane,^8^ or inclusion of functional groups that most likely enhance the association with the plasma membrane.^9^ Nevertheless, all of these import strategies are restricted to proteins and small molecular weight cargo. For oligonucleotides (ONs), delivery occurs through endocytosis in all cases reported so far. Delivery of ONs, ranging from antisense ON to plasmid DNA, is a further important application of CPPs.

CPPs thus are a highly interesting modality within the spectrum of materials for oligonucleotide delivery, belonging to the wider group of polymer-based delivery systems.^10^ At present, lipid nanoparticles (LNPs) are the most widely used and explored delivery vehicles. The SARS-Cov-2 vaccines are LNP formulations, and also mRNA formulations in clinical studies for protein replacement therapy are based on LNPs.^11,12^ However, LNPs suffer from limited stability, contain multiple different lipids, and require microfluidic mixing devices and separation of organic solvent. Thus, there is still an unmet need for new formulation modalities that are simpler in structure, biodegradable into endogenous compounds, and easier to formulate. Peptide-based biomaterials are promising candidates to fulfill these requirements.

For CPPs, the formulation can be through direct covalent conjugation with the CPP for ON analogs that do not have a negative charge in their backbone.^13^ For all negatively charged ONs, formulation occurs through noncovalent complexation of the ONs with the positively charged peptides. Uptake of these polycationic complexes (polyplexes) is a multistep process that occurs through association with the negatively charged glycocalyx on the cell surface, followed by induction of endocytosis, capture inside endosomal vesicles, and ultimately endosomal release.^14^ Alternatively, scavenger receptor-mediated endocytosis has been proposed.^15^ Whereas basically all positively charged CPPs possess the capacity to form nanoparticles with negatively charged ONs, the resulting polyplexes vary substantially in inducing endosomal uptake. Endosomal release is the critical last step for successful delivery, and only a few peptides induce this process efficiently.^16^

PepFect14 (PF14) is a peptide that yields efficient cytosolic delivery for various ONs in vitro and in vivo.^17−21^ The peptide is amphipathic in nature and is *N*-terminally stearylated. PF14 is derived from PepFect3 (PF3), an analog of Transportan 10 (TP10),^22,23^ with four out of five lysine residues replaced by the nonproteinogenic amino acid ornithine. Polyornithines outperform polylysines in transfection efficiency, which was attributed to a tighter association with the ON.^24^ Moreover, in PF14, all isoleucines present in PF3 were replaced with leucines to reduce steric repulsion between the cargo (ON) and peptide.^25^ At higher concentrations, however, PF14 is also cytotoxic through its membrane-active nature, which results from its amphipathic character.

Next to membrane activity, the proton-sponge effect is the most widely employed concept to enhance endosomal escape.^26^ This concept is based on the acidification-dependent protonation of slightly basic groups, which leads to a further import of protons with a concomitant influx of chloride ions resulting in an increased osmotic pressure ultimately causing rupture of the endosomal membrane and thus the cytosolic release of the ON. For peptides, histidine side chains are incorporated for this purpose. For oligoarginines and other CPPs, linkage into longer polymers via disulfide linkage has also been shown to increase delivery activity.^27,28^ This result aligns with other polycationic polymers, such as polyethylenimine, for which polymer length correlates with activity.^29^ Also, reducible linkages have been demonstrated to benefit both pDNA^30^ and messenger RNA (mRNA)^31−33^ delivery. Several mechanisms have been attributed to the enhanced transfection efficiency of cysteine/disulfide-polymerized carriers, such as rapid disulfide cleavage by cytoplasmic thioredoxin reductases and glutathione,^34,35^ which results in rapid release of nucleic acid from its carrier, and increased uptake via disulfide exchange on the cell surface.^36^

We are unaware that the capacity of these various structural concepts to yield cellular uptake and cytosolic delivery of ON has been explored in one common structural framework. The 21-amino-acid CPP, derived from the *N*-terminal domain of human lactoferrin (hLF), shares characteristics of arginine-rich CPPs and fails to deliver ONs into the cytosol.^16,37^ The peptide shows an interesting structure–activity relationship, requiring cyclization by an intramolecular disulfide bond for activity.^37^ We have shown before that the peptide provides an interesting scaffold to investigate structural principles of CPP activity.^38^

Here, we used the hLF peptide to implement the above-mentioned structural principles in a stepwise manner. First, we generated linear peptide oligomers through intermolecular disulfide bonds instead of intramolecular disulfide bond formation. Second, we introduced histidine and ornithine residues to explore the relevance of the proton sponge effect and ornithine residues for activity. Finally, the membrane activity was enhanced through the incorporation of amphipathic structural motifs from PF14. Ultimately, membrane activity was the only structural principle conferring a significant delivery benefit.

## Results

### Cellular Uptake of hLF and PF14 mRNA Polyplexes

**Figure 1:**
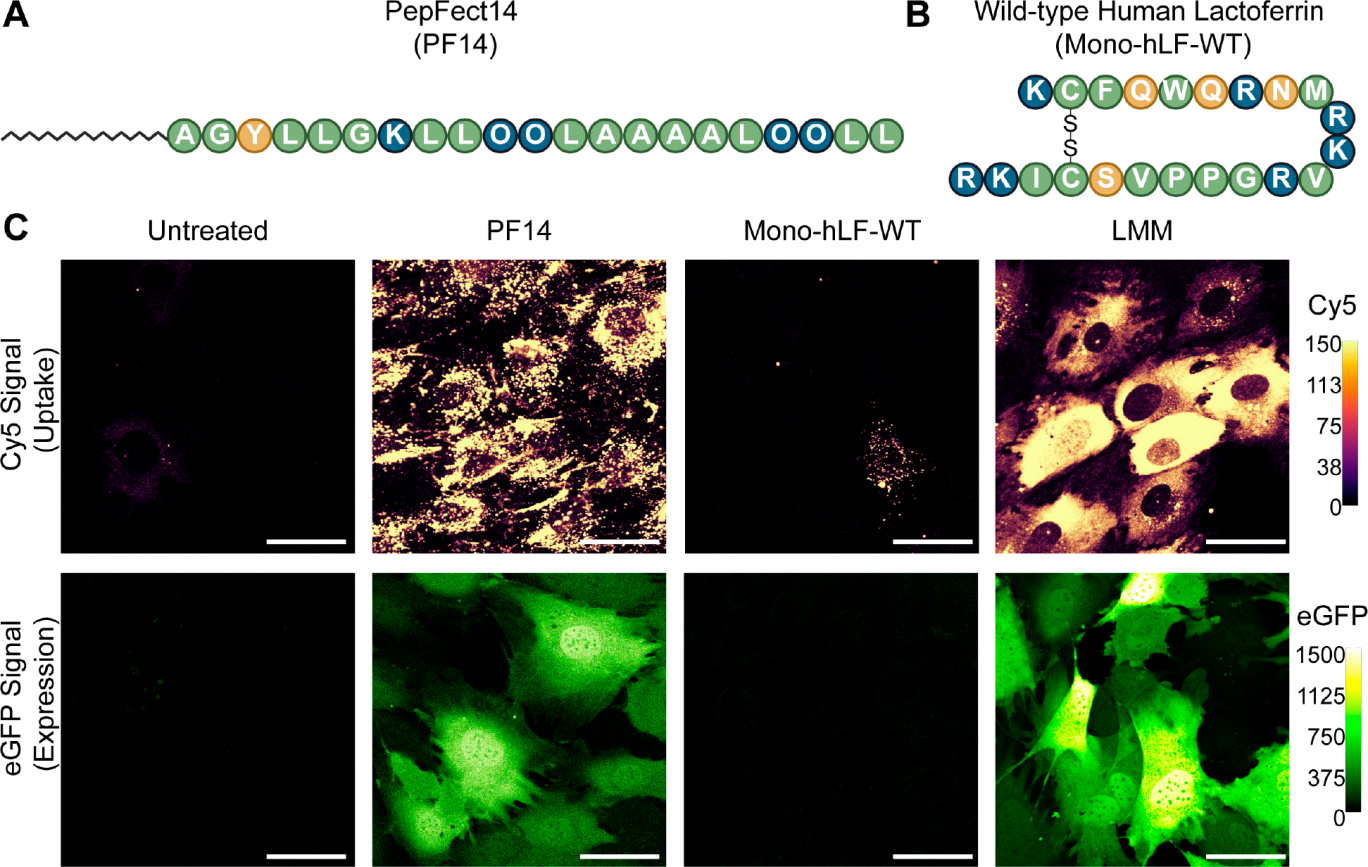
(A) Structures of PepFect14 and (B) Mono-hLF-WT and comparison of (C) uptake (top) and transfection efficiencies (bottom). As a positive control, transfection with Lipofectamine MessengerMAX (LMM) is included. LMM eGFP was acquired with a 10-fold lower gain. Cellular uptake was detected with Cy5-labeled mRNA and protein expression with unlabeled mRNA because of higher expression efficiency. Data are representative of five independent experiments. Scale bars represent 50 μm. O denotes the nonproteinogenic amino acid ornithine. Brightness and contrast were equally adjusted across conditions per fluorophore, according to the calibrated look-up table (right) where the values reflect pixel intensities.

We have demonstrated before that PF14-mRNA polyplexes show efficient cellular uptake and cytosolic delivery, thereby yielding protein expression.^20,39,40^ For hLF, we have previously addressed delivery of siRNA and antisense oligonucleotides for which the peptide failed to yield down-regulation of the target mRNA.^16,21^ Therefore, we first compared both peptides with respect to cellular uptake and delivery of mRNA. At an N/P ratio of 3, PF14 and the monomeric wild-type hLF (Mono-hLF-WT), which was oxidized at conditions that favor intramolecular disulfide bond formation, readily formed monodisperse nanoparticles with mRNA with diameters of 47.9 ± 1.18 nm and 115 ± 1.03 nm (Table S2), respectively. Polyplexes were formed either with Cy5-labeled enhanced green fluorescent protein (eGFP) mRNA to monitor cellular uptake 2 h post-transfection or with eGFP mRNA to detect protein expression 24 h post-transfection. Whereas PF14 (Figure 1A) yielded strong cellular uptake and also protein expression across all cells, for hLF (Figure 1B), only little uptake and no expression were present (Figure 1C). We also included Lipofectamine MessengerMAX (LMM) as a further reference. Compared to PF14, mRNA fluorescence was homogeneously distributed throughout the cytosol with no signals present in nuclei, consistent with previous observations.^41^ This result demonstrates that also for PF14, a significant fraction of material remains associated with vesicular structures. In addition, the sparing of the nuclei from fluorescence also demonstrates that the mRNA reaches the cytosol in an intact form.^42^

### Design of Structural hLF Variants to Delineate Relevant Characteristics of the Structure–Activity Relationship

**Figure 2:**
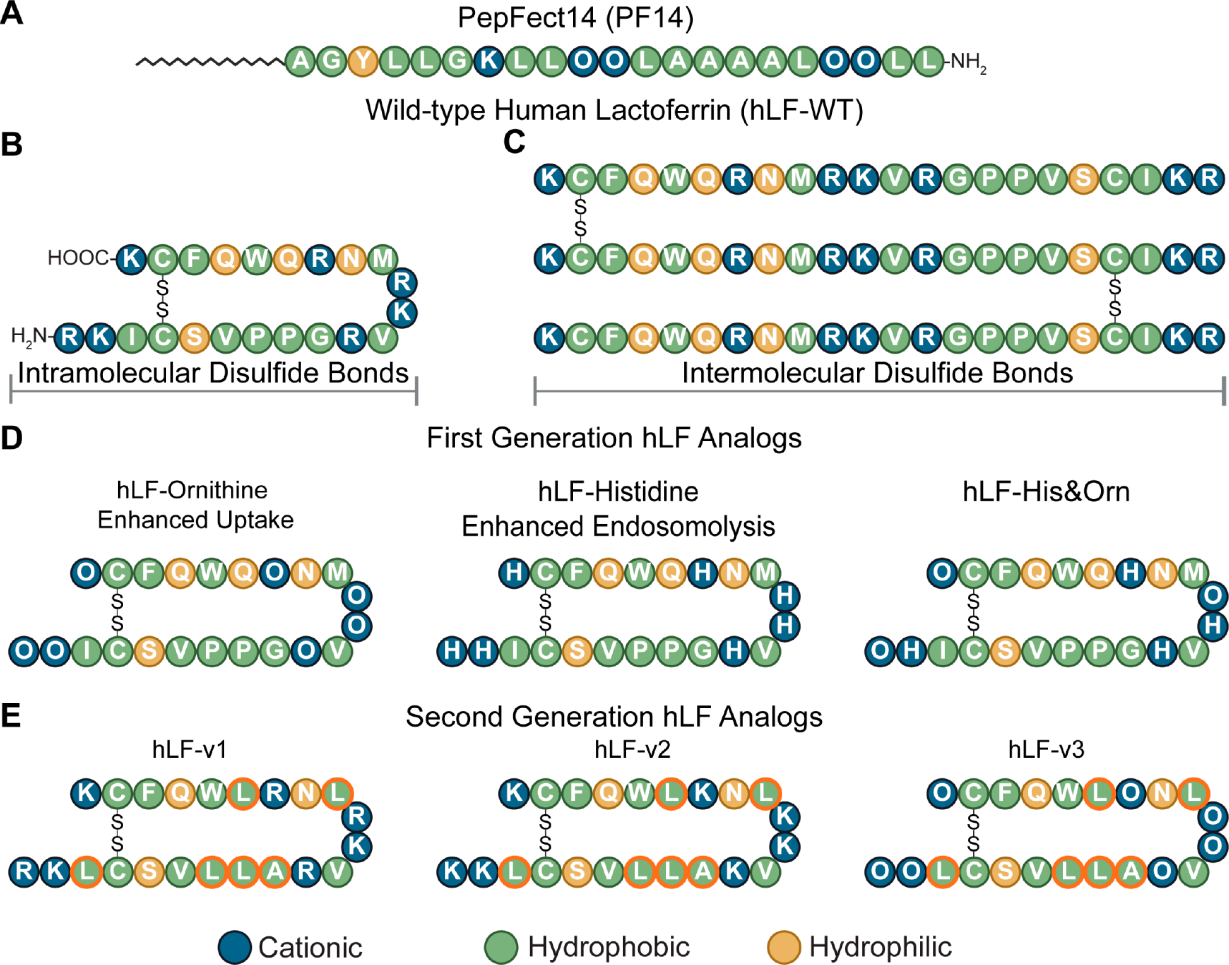
Variants to identify the relevant structural characteristics that are decisive for the activity of peptide-mediated mRNA delivery. (A) Structure of PF14, (B) structure of hLF with intramolecular disulfide bond (Mono-hLF-x), (C) oligomerization of hLF via intermolecular disulfide bond formation (Poly hLF-x)—oligomerization can occur head to tail, tail to tail, or head to head with no directionality. (D) hLF analogs with replacement of positively charged residues for ornithines and histidines, (E) hLF analogs with amphipathic PF14 structural motifs (delineated in orange). O denotes the nonproteinogenic amino acid ornithine.

After demonstrating the considerable differences in uptake and cytosolic delivery, we considered the peptides PF14 (Figure 2A) and the monomeric-hLF-WT (Figure 2B) at the two ends of the structure–activity space of good uptake and delivery and poor uptake and delivery. This starting point allowed us to incorporate structural features into the hLF peptide, which have all been associated with enhanced delivery activity, and assess their potency in one coherent context. These structural features are comprised of oligomerization through intermolecular disulfide bonds (poly hLF-x; Figure 2C), exchange of positively charged residues for ornithines (Figure 2D), and incorporation of histidines for endosomal release via the proton sponge effect and finally the incorporation of amphipathic structural motifs from PF14 (Figure 2E).

### Impact of Oligomerization on mRNA Uptake and Delivery

**Figure 3:**
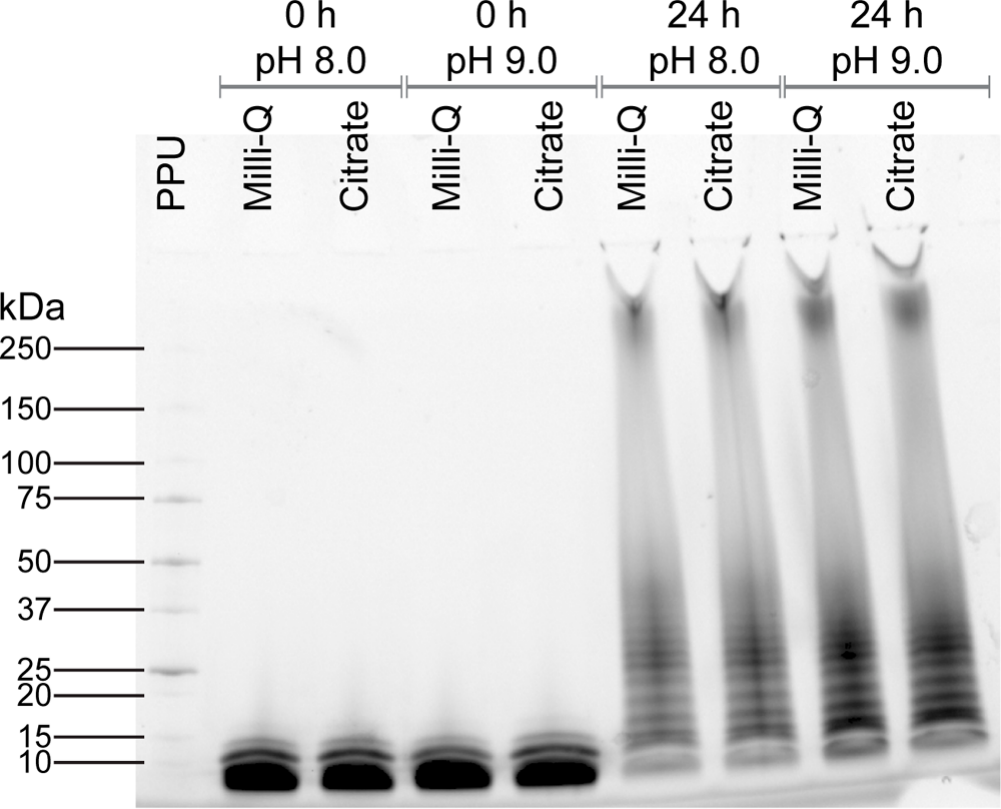
Oligomerization of hLF-WT peptides into higher-order oligomers. In the left four lanes, peptides were incubated at a concentration of 5 mM, for the right four lanes at 50 mM for the indicated times and pH, followed by dilution in deionized water or citrate buffer pH 5. Protein concentrations were equalized across conditions, and samples were run on a stain-free gel for direct visualization. PPU: Precision Plus Protein Unstained Protein Standards. Data are representative of three independent experiments.

Various methods have been described for disulfide-mediated oligomerization of peptides, including dimethyl sulfoxide-driven oxidation^28^ and substrate-initiated polymerization starting from intramolecular disulfides.^43^ We opted for pH- and concentration-dependent polymerization in an aqueous buffer. Disulfide formation requires a slightly basic pH, and disulfide exchange is strongly reduced at acidic pH.^44,45^ Disulfide bond formation was carried out at either pH 8 or pH 9 with a peptide concentration of 5 mM for intramolecular disulfide bond formation or 50 mM for intermolecular disulfide bond formation. After incubation, mono-hLF samples were diluted in MQ water. Notably, all poly-hLF species formed insoluble gels over 24 h of incubation at 37 °C, supporting oligomer formation. These gels were redissolved and diluted using a citrate buffer of pH 5.0 to a concentration of approximately 25 mM. The oligomerized species had a length of >10 monomers (i.e., >25 kDa; Figure 3). There was no difference between pH 8 and pH 9. However, all subsequent 24 h polymerizations were performed at pH 9 to ensure robust conditions.

**Figure 4:**
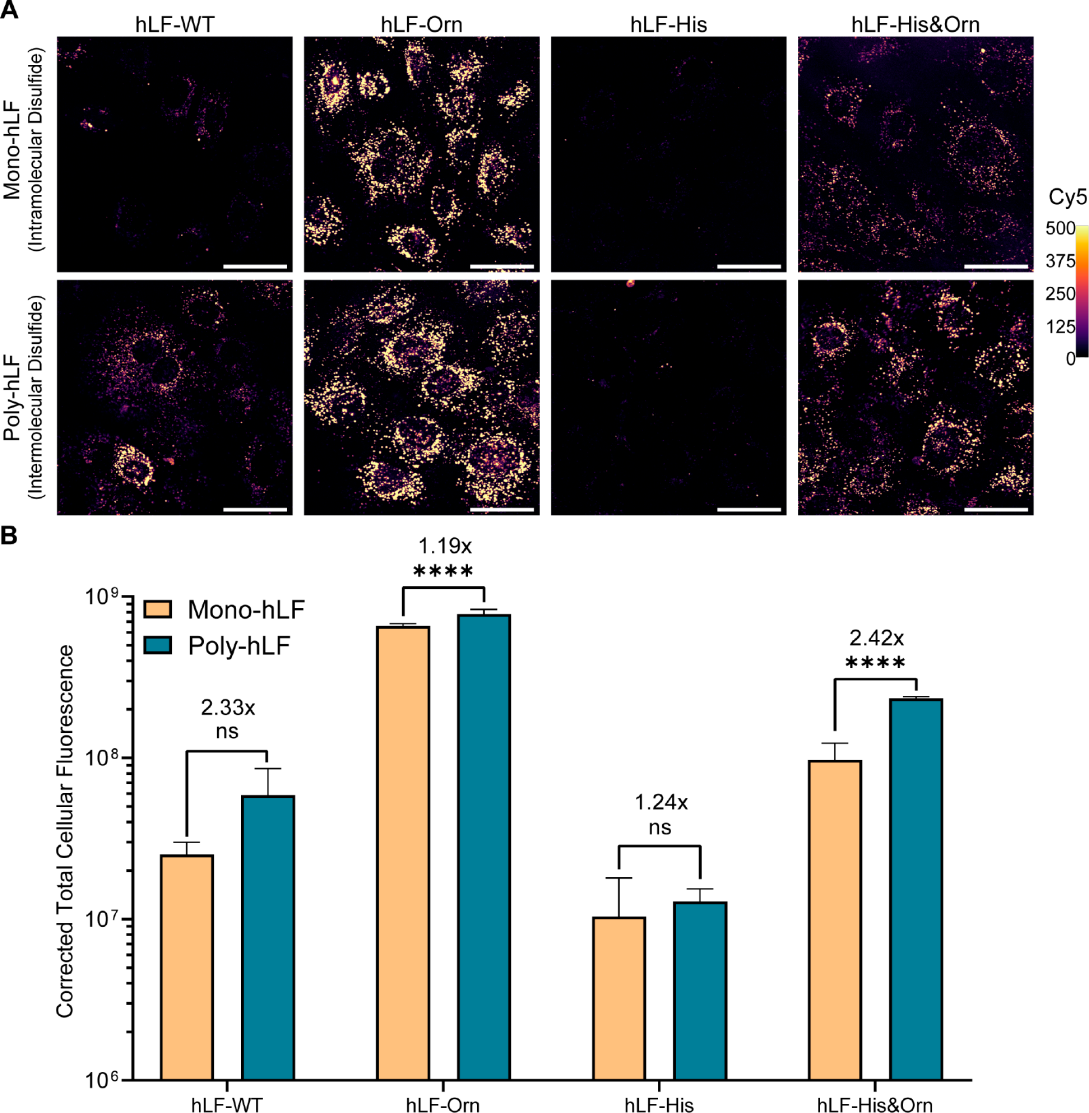
Impact of oligomerization and ornithine/histidine exchange on uptake efficiency. (A) Live-cell confocal microscopy of MC3T3 cells incubated with Cy5-eGFP mRNA polyplexes formed from either monomeric or polymerized peptides 2 h post-transfection. (B) Quantification of Cy5 signal, *n* = 5 (field of view) per condition. Data are representative of three independent experiments. Data represent the mean + SD. Scale bars represent 50 μm. ns: nonsignificant; ****p ≤ 0.0001. The numbers above bars indicate the ratio of Poly hLF-x vs Mono-hLF-x. Brightness and contrast of Cy5 were equally adjusted across conditions, according to the calibrated look-up table (right) where the values reflect pixel intensities.

For the ornithine and the histidine/ornithine-substituted analogs, oligomerization significantly increased uptake efficiencies by factors of 1.2–3 (Figure 4A,B). For the wild-type sequence, the difference did not reach significance due to larger variations between the individual images. For the histidine-substituted variant, poor uptake was only slightly improved (∼25%) by oligomerization. We attribute this lack of activity to poor stability of the polyplexes due to weak complexation with the weakly basic histidine residues. By comparison, uptake of the ornithine-substituted variant exceeded the one of the wild-type peptide by about a factor of 20, followed by the histidine/ornithine-substituted variant. Furthermore, more oligomerization occurred at low concentrations for the ornithine variant, whereas the histidine variant showed a reduced oligomerization propensity (Figure S2).

### Incorporation of PF14 Structural Elements into the hLF Backbone

Next, we explored the incorporation of amphipathic features into the hLF backbone. At the same time, we asked whether, in the amphipathic context, the nature of the positively charged residue influenced activity. Whereas in hLF, arginine is the prominent charge carrier, in PF14, this is ornithine. For CPPs, the bidentate nature of the guanidino group of arginines has been associated with increased cellular uptake.^14^

We used helical wheel projections and average hydrophilicity calculations^46^ to aid the rational design of amphipathic hLF variants (Figure S3). Considering the ratio of hydrophobic residues as a percentage of total residues, PF14 has 24% with a hydrophilicity score of −0.20, whereas hLF-WT has 50% and 0.36, respectively. Ultimately, the hLF-WT sequence was altered by changing one glutamine, methionine, two prolines, and one isoleucine to leucines and one glycine to alanine. Although the hydrophobic residue to total residue ratio only decreased by 5% for the amphipathic hLF variants compared to hLF-WT, the hydrophilicity score was 6-fold lower (0.06).

**Figure 5:**
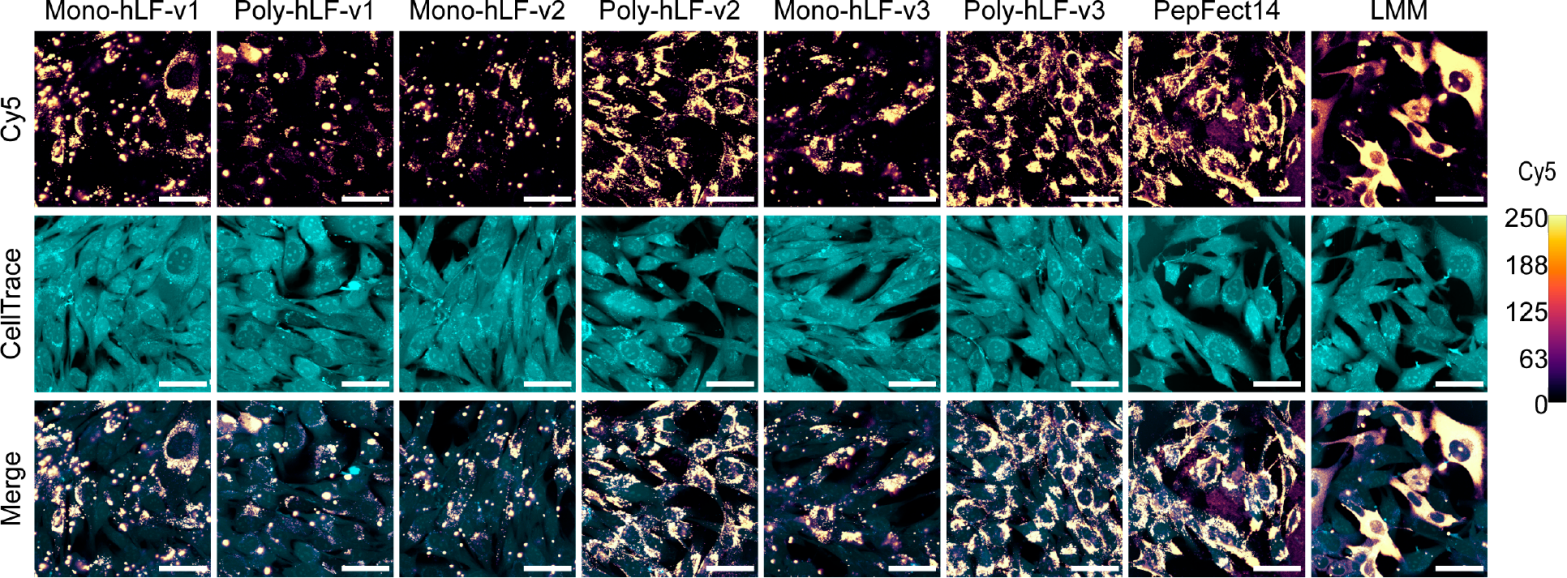
Incorporation of PF14 structural elements into hLF improves uptake efficiency in a polymerization-dependent manner. Cells were incubated with polyplexes containing Cy5-labeled mRNA for 2 h, followed by live-cell confocal microscopy. Data are representative of four independent experiments. Scale bars represent 50 μm. Brightness and contrast of Cy5 were equally adjusted across conditions, according to the calibrated look-up table (right) where the values reflect pixel intensities. Quantifications of uptake are shown in Figure S6.

The amphipathic hLF variants also showed oligomerization, however, for variants v2 and v3 no clear pattern of individual bands was visible (Figure S4). Surprisingly, of all three variants, hLF-v1, which has the same cationic amino acids as hLF-WT, performed the worst and tended to aggregate upon polymerization (Figure 5). It should be noted that this aggregation was only revealed by confocal microscopy, as particle characterization with dynamic light scattering (DLS) indicated a monodisperse particle size of 72 ± 2.1 nm (Table S2). The discrepancies between these particle sizes are most likely due to the presence of serum in the microscopy experiment. For the lysine-substituted peptide (hLF-v2), oligomerization yielded a pronounced increase in uptake, as was the case for the ornithine-substituted (hLF-v3) one. The uptake of the polymerized ornithine-substituted peptide was comparable to the one of PF14. By comparison, hLF-v1 polymerization showed no benefit for uptake (Figure S5).

### Delivery of mRNA by hLF Variants

Cellular uptake is insufficient as a predictor for cytosolic delivery.^47^ Therefore, we formulated mRNA coding for luciferase with the different hLF variants and determined reporter protein expression. We opted for luciferase instead of eGFP as it allows more robust quantitative comparisons across a larger dynamic range. Polyplexes were formulated at N/P ratios of 3 and 5, as higher N/P ratios may promote increased polyplex formation, stability, and uptake efficiency.

**Figure 6:**
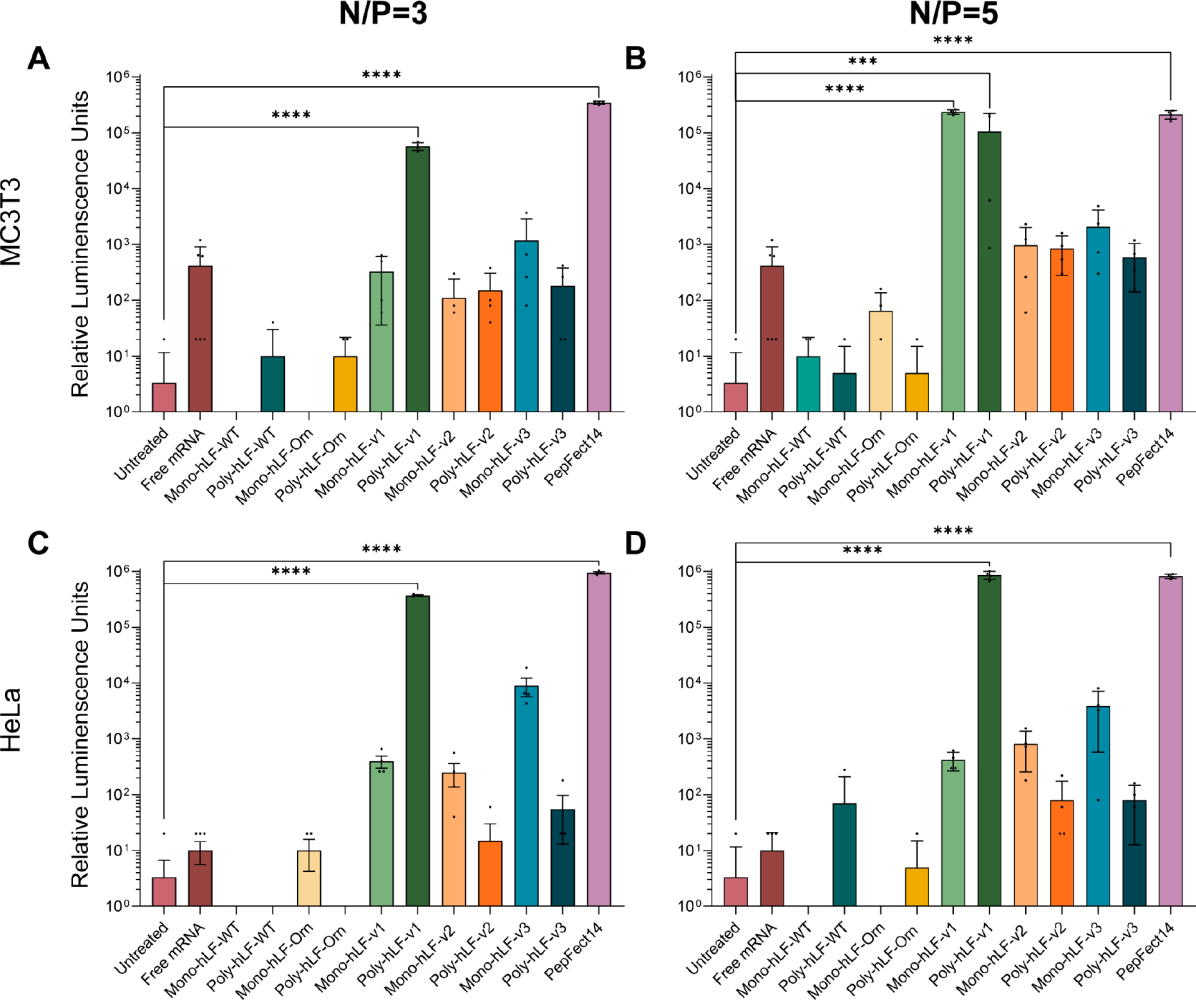
Activity of monomeric and polymeric hLF variants in mRNA delivery as measured by luciferase expression at nitrogen over phosphate ratios (N/P) of 3 or 5. (A) Luciferase expression in MC3T3 cells at an N/P of 3 (B) and an N/P of 5. (C) Luciferase expression in HeLa cells at an N/P of 3 (D) and an N/P of 5. Data represented as mean ± SD of technical quadruplicates. Note that the untreated and free mRNA conditions are identical for an N/P of 3 and an N/P of 5. All conditions were compared to untreated, and only significant differences are depicted. *p ≤ 0.05, **p ≤ 0.01, ***p ≤ 0.001, and ****p ≤ 0.0001.

At an N/P ratio of 3, significant activity was observed for the polymeric arginine-containing variant (poly hLF-v1), albeit 6-fold lower than PF14 (Figure 6A). Interestingly, at an N/P of 5, activity was also observed for the monomeric (mono-hLF-v1) as well as for the polymeric species (poly hLF-v1; Figure 6B), with the former yielding an expression ∼10% higher than PF14, while the latter only reached approximately half of PF14-induced luciferase expression. Interestingly, despite the tendency to aggregate, hLF-v1 outperformed the other two variants with amphipathic motifs, which showed only about 1% of the activity, independent of polymerization. By comparison, the hLF-WT and the histidine and ornithine variants showed no activity. Luciferase activity was even lower than those for cells incubated with naked mRNA, indicating that complexation with these peptides shielded the mRNA from uptake and intracellular delivery. These observations were independent of the N/P ratio.

Additionally, we gauged the impact of different mRNA polyplexes on the metabolic activity of cells. For MC3T3 cells, no significant negative impact of mRNA transfection was found at an N/P of 3 with all conditions ranging between 95 and 110% relative metabolic activity, except for Mono-hLF-WT and Mono-hLF-v3, which both had an activity of 125% (Figure S6A). At an N/P of 5, mono-hLF-v2 and poly hLF-v3 reached ∼125% metabolic activity, whereas the higher charge ratio of PF14 significantly decreased metabolic activity to 64% (Figure S6B).

To validate the robustness of our results, we also transfected HeLa cells. At an N/P of 3, transfections with either poly hLF-v1 or PF14 resulted in significant luciferase expression, with PF14 resulting in ∼2.5 times higher expression (Figure 6C). Once again, at an N/P of 5, the poly hLF-v1 yielded slightly higher expression (∼5%) than PF14 (Figure 6D). The same observations were made for the other peptides, as for MC3T3 cells. Regarding metabolic activity, Hela cells were more sensitive to PF14 than MC3T3 cells, with a significant decrease of activity to 72% (Figure S6C). This increased sensitivity became even more apparent at an N/P of 5, where PF14 transfections reduced the metabolic activity to 13% and mono-HLF-WT to 83% (Figure S6D). Crucially, it should be noted that all conditions still had intact, confluent monolayers without clear indications of massive cell death.

Overall, hLF polyplexes formed at an N/P of 5 tended to show higher activity than polyplexes formed at an N/P of 3. Moreover, hLF variants with high N/P ratios did not show reduced metabolic activity. Conversely, a higher charge ratio negatively impacted both cell lines’ mRNA expression and metabolic activity for PF14. As an orthogonal line of evidence, we formed polyplexes with (Cy5-) eGFP mRNA at an N/P of 3. We confirmed the successful delivery of both eGFP-coding mRNAs with poly-hLF-v1 with live-cell confocal microscopy (Figure S7).

### Resistance of Polyplexes against Heparin-Driven Decomplexation

Following the demonstration of enhanced cellular uptake, we finally aimed to clarify the structural differences of the various polyplexes. For this purpose, we performed heparin-driven decomplexation assays. Heparin is a negatively charged polysaccharide that can competitively displace the mRNA from the polyplex. Structurally, heparin is similar to the negatively charged proteoglycans on the cell surface. Whereas these molecules have also been discussed as receptors for the uptake of polyplexes,^48^ they could also induce polyplex dissociation before cell entry.

**Figure 7:**
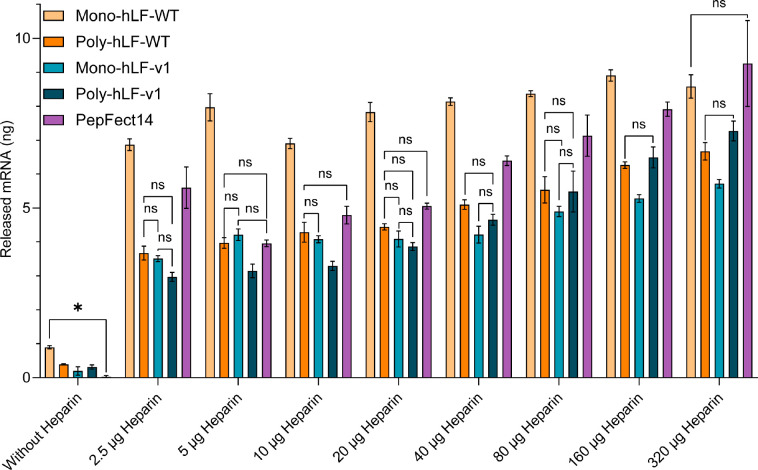
Introduction of hydrophobic residues and polymerization stabilizes polyplexes against heparin-induced decomplexation. All nanoparticles were formed at an N/P of 5. Data representative of three independent experiments and represented as the mean ± SD of three technical replicates. Statistics were performed per heparin condition, and except for the conditions without heparin, only nonsignificant differences are depicted. All significant differences had *p* values ≤ 0.0147, except for Mono-hLF-WT vs PF14 without heparin conditions, which had a *p* value of 0.0252.

For conditions without heparin-mediated decomplexation, only the mono-hLF-WT showed a significantly (*p* = 0.0252) worse encapsulation efficiency than PF14 (Figure 7). Additionally, heparin decomplexation of mono-hLF-WT NPs revealed significantly lower stability of these polyplexes compared to PF14, except for the highest heparin dose. Disulfide-mediated polymerization of hLF-WT increased the heparin resistance of polyplexes, as evidenced by the significantly lower release of mRNA of polymeric hLF-WT as opposed to its monomeric counterpart (mono-hLF-WT). Incorporating amphipathic features into the hLF sequence stabilized the polyplexes regardless of polymerization state (monomeric vs polymeric). Overall, integrating the structural features of PF14 into hLF or the disulfide-mediated polymerization of hLF yielded polyplexes significantly more resistant to heparin-mediated decomplexation than PF14.

As the luciferase experiments revealed a role of the N/P ratio on transfection efficiency (Figure 6), we opted to test a range of N/P ratios with three different peptides, with either low or high doses of heparin (Figure S8). At an N/P of 1 and 3, no difference was present between poly-hLF-Orn or poly-hLF-v1 when coincubated with either low or high heparin amounts. However, at an N/P of 5, both hLF species displayed more resistance to heparin-mediated decomplexation than PF14. When increasing the charge ratio to an N/P of 7, at a low heparin dose, PF14 was least stable. In contrast, at a high heparin dose, PF14 displayed similar decomplexation behavior as both poly-hLF-Orn and poly-hLF-v1. The conditions without heparin revealed differences in the complexation behavior of the different peptides. For PF14, over 90% of the initial mRNA dose was complexed regardless of charge ratio. For hLF peptides, encapsulation efficiencies over 60% were only observed at an N/P of 5 or higher.

## Discussion

Despite intense research, only a few CPPs efficiently deliver ONs into the cytosol at a favorable activity/toxicity balance. PF14 is one such peptide. The peptide features an *N*-terminal stearylation, ornithine residues as positive charge carriers, and an amphipathic character through the distribution of positively charged and leucine residues. The stearylation confers increased stability of polyplexes compared to the nonstearylated analog TP10.^16^ Other features that have been associated with the increased delivery activity of peptides are histidines,^49−51^ which act via the proton sponge effect and polymerization through disulfides. Next to the stabilization,^28^ disulfides can increase uptake via disulfide exchange on the cell surface.^36^

Using the human lactoferrin-derived CPP hLF as a scaffold, we explored the stepwise incorporation of structural features associated with an enhanced delivery activity. Incorporating histidines as positive charge carriers alone did not yield stable polyplexes, most likely because protonation is insufficient at neutral pH. By comparison, hLF analogs that contained both histidines and ornithines yielded uptake that outperformed the wild-type peptides. The enhanced uptake of the ornithine-substituted variants is consistent with earlier reports that ornithines yield a tighter association of the carrier with the ONs.^24^ Only when aliphatic residues or ornithines were integrated into the hLF structural scaffold was the uptake comparable to the one of PF14. Nevertheless, the resulting hLF analog was still far from a structural mimetic of PF14. Eight out of 21 amino acids still corresponded to the original hLF sequence, and the distribution and pattern of leucine residues differed for the two peptides. Thus, our results clearly demonstrate that the specific functional characteristics of ornithine as a positive charge carrier and of an amphipathic structure are also operational within a different structural context. Notably, ornithines and amphipathic structure yielded more cellular uptake and endosomal release of polyplexes compared to hLF.

Interestingly, protein expression did not fully correlate with mRNA uptake. The lysine- and ornithine-substituted variants showed little activity, and the arginine-substituted one, hLF-v1, showed the highest activity. This activity was accompanied by aggregation of the polyplexes when in contact with cells. Apparently, the membrane-active hLF variants still required high local doses to yield sufficient mRNA delivery. This hypothesis is supported by the fact that, for hLF-v1, fewer cells were positive for eGFP expression than for PF14 (Figure S7). As a technical note, it is remarkable that for cells treated with naked mRNA, activity was up to 100 times higher than the background. These data indicate that, when using highly active mRNA, this condition needs to be included as an additional control. We can only speculate that protein expression results from a few cells with compromised membrane integrity. However, we observe that variation between conditions yielding only low protein expression is higher between technical replicates than for conditions yielding high protein expression.

The combination of positive charge next to aliphatic residues is also present in a series of antimicrobial histidine-rich peptides that showed DNA delivery activity.^52^ Unfortunately, this study did not address the importance of the histidine residues for activity. The heparin displacement assay indicated that the stability of polyplexes against dissociation by negatively charged polymers is an important determinant of uptake efficiency. Interestingly, incorporating amphipathic sequence motives into hLF stabilized hLF-v1 polyplexes regardless of polymerization state (inter- vs intramolecular disulfides). PF14 is a stearylated analog of the CPP TP10.^53^ Stearylation yields micelle formation, and polyplexes formed from stearylated TP10 analogs are more stable against decomplexation than TP10 itself.^16^ Our data clearly demonstrate disulfide-mediated polymerization as an alternative means for stabilization.

We should note that the incorporation of histidines and disulfide-mediated polymerization were successful in the hands of others. Lo and Wang introduced histidine residues and cysteines into the Tat peptide, which strongly increased activity.^49^ However, in their case, transfections were performed in serum-free conditions, which is a very different environment with respect to polyplex stability and uptake, usually yielding stronger uptake than for serum-containing conditions. Also, N/P ratios were tested that far exceeded the ones we used. Using super-resolution microscopy, we have shown that at N/P ratios >3 most material is only loosely associated with polyplexes or present as free peptide.^54^ These results are corroborated by DLS characterization, where polyplexes formed at an N/P of 5 consistently show larger hydrodynamic radii and higher polydispersity (Table S2).

## Conclusion

Our data underline the presence of overarching principles for delivery vehicles to show activity. Importantly, membrane activity is an indispensable ingredient for delivery. The CPP polyplexes achieve this membrane activity by virtue of the amphipathic structural motifs, lipid nanoparticles by disintegration within the endosomal compartment and membrane activity of the incorporated ionizable lipids.^55^ And also for polymer-based systems, lipophilicity enhanced activity.^55,56^ Simultaneously, nanoparticles must be sufficiently stable to withstand serum and dissociation by the glycocalyx on the cell surface. In the case of the peptides investigated in this study, stability can be achieved either through micelle formation or through linkage into polymers. However, as PF14 still outperformed also the most polymeric hLF variants, we conclude that micelle formation is superior as observed for other polymeric systems.^57^ Moreover, the lack of delivery activity for polymeric hLF-WT for which polyplexes are as stable as for PF14 demonstrates that stability is a necessary but not sufficient characteristic. So far, we have not been able to demonstrate delivery activity for any delivery vehicle that has arginine residues in a nonmembrane-active context, such as nona-arginine, hLF, and Tat, even if additional features such as oligomerization were included.

Importantly, this conclusion means that peptide-mediated delivery obeys very similar structural principles as delivery through lipid-based nanoparticles, for which partial disintegration along the endolysosomal pathway also yields membrane-disrupting activity.^58^

## Materials and Methods

### Cell Culture

Subconfluent cultures of the MC3T3-E1 subclone 4 (CRL-2593, American Type Culture Collection; ATCC, Manassas, VA, USA) preosteoblastic murine cell line were maintained in Minimal Essential Medium α (MEM-α; Gibco, Waltham, MA, USA, Cat. No. A10490-01), supplemented with 10% v/v fetal bovine serum (FBS; Gibco, Cat. No. 10270-106), which will be referred to as complete medium. All experiments were performed with MC3T3 cells with a passage number lower than 25.

Subconfluent cultures of the HeLa human cervical adenocarcinoma cell line (DSMZ no. ACC57, Leibniz Institute DSMZ-German Collection of Microorganisms and Cell Cultures, Braunschweig, Germany) were maintained in RPMI 1640 Medium (Dutch modification, Gibco Cat. No. 22409031) supplemented with 10% v/v FBS and GlutaMAX (Thermo Fisher Scientific, Waltham, MA, USA, Cat. No. 35050038). All experiments were performed with HeLa cells with a passage number lower than 15.

### Messenger RNA (mRNA)

Enhanced green fluorescent protein (eGFP) mRNA (L-7601) and 5-methoxyuridine-substituted cyanine 5 (Cy5)-labeled eGFP mRNA (L-7701) were purchased from Trilink Biotechnologies (San Diego, CA, USA). mRNA coding for secreted nanoluciferase (SecNLuc) and uncapped SecNLuc mRNA, used for heparin decomplexation assays, were purchased from RIBOPRO (Oss, The Netherlands). All mRNA was aliquoted at 100 ng μL^–1^ in nuclease-free water (Thermo Fisher Scientific) in DNA LoBind tubes (Eppendorf, Hamburg, Germany), snap-frozen in liquid nitrogen and stored at −80 °C until use. Before use, the mRNA solutions were thawed and kept on ice.

### Cell-Penetrating Peptides

Peptides were purchased from EMC microcollections (Tübingen, Germany). The identity and purity of the peptides were determined by mass spectrometry and reversed-phase high-performance liquid chromatography by EMC microcollections. An overview of all peptide sequences, molecular weights, and modifications is provided in Table S1 and Figure S1. All peptides were dissolved in Milli-Q (MQ) water, stored in Protein LoBind tubes (Eppendorf, Hamburg, Germany), and incubated at room temperature (RT) for 20 min under gentle agitation before aliquots were snap-frozen in liquid nitrogen and stored at −20 °C.

### Oxidation of hLF Peptides

For the intramolecular oxidation of hLF peptides, peptides were dissolved to 5 mM in 50 mM 4-(2-hydroxyethyl)-1-piperazineethanesulfonic acid (HEPES; Cat No. H4034, Sigma-Aldrich, St. Louis, MO, USA) buffer, at pH 8.0. For intermolecular disulfide bond formation, peptides were dissolved in 50 mM HEPES at pH 9.0 at a final peptide concentration of 50 mM. After dissolution, the pH was adjusted with 0.1 M NaOH. In either case, 30 μL of peptide solution was added to polypropylene tubes (Greiner Bio-One, Kremsmünster, Austria, Cat No. 673210) and incubated in a T300 thermocycler (Biometra, Göttingen, Germany) for 2 h at 37 °C for the formation of intramolecular disulfide bonds (mono-hLF), and for 24 h at 37 °C for the formation of intermolecular disulfide bonds (poly hLF). Samples were diluted in 20 mM citrate buffer at pH 5.0 or MQ water to stabilize the formed disulfide bonds.

### Sodium Dodecyl-Sulfate Polyacrylamide Gel Electrophoresis (SDS-PAGE)

Four microliters of 5 mM solutions of hLF species were mixed with 10 μL of 4x Laemmli buffer (Bio-Rad, Hercules, California, USA) and 26 μL of MQ water to assess the oxidation of hLF peptides. Without a boiling step, samples were added to a 4–15% Mini-PROTEAN TGX Stain-Free Protein Gel (Bio-Rad, Cat. No. 4568084 or 4568086). The outer buffer chamber was filled with tris(hydroxymethyl)aminomethane (tris)-glycine buffer at pH 8.5, with final concentrations of 25 mM of Tris and 200 mM of glycine. The tris-glycine buffer was supplemented with 1% w/v SDS for the inner buffer chamber. As a reference for molecular weight, 2 μL of Precision Plus Protein Unstained Protein Standards, Strep-tagged recombinant (Bio-Rad, Cat No. 1610363), was included for each gel. Gels were run at a constant voltage of 200 V for approximately 30 min using a Bio-Rad electrophoresis unit (PowerPac 3000). Stain-free protein detection was performed with the Gel Doc EZ system and Stain-Free sample tray (BioRad, Cat No. 1708274). Gels were activated by UV light while using the default settings for best sensitivity.

### mRNA Nanoparticle Formation

Peptide-mRNA polyplexes were formulated as previously described.^40,41^ In short, two separate stock solutions of mRNA and peptide were prepared in MQ and simultaneously dispensed with electronically dispensing pipettes (E4 Electronic Pipette, LTS E4-100XLS+, Mettler-Toledo Rainin, LLC, Oakland, CA, USA) at a flow rate of 11 mL min^–1^. All polyplexes were formed at a concentration of at least 10-fold the final intended concentration for transfections.

For the formation of cationic lipid-based complexes (lipoplexes), Lipofectamine MessengerMAX (LMM; Thermo Fisher Scientific) was used according to the manufacturer’s instructions. In short, LMM was incubated in Opti-MEM (Gibco, Cat. No. 11058021) for 10 min at room temperature (RT). The appropriate amount of mRNA solution was diluted in Opti-MEM and incubated with LMM for at least 5 min at RT.

The hydrodynamic diameter of the nanoparticles was measured at 25 °C by dynamic light scattering (DLS) using a Zetasizer Nano ZS (Malvern Instruments, Worcestershire, UK) equipped with a 4 mW He–Ne laser (633 nm) with a backscatter detection angle of 173°. At least 40 μL of 10 times concentrated nanoparticle solution was measured in a UV-cuvette (BrandTech Scientific, Essex, CT, USA, Cat. No. 759200).

### mRNA Transfections

One day before mRNA transfections, 10 000 MC3T3 cells in 100 μL were seeded in 96-well plates (Greiner Bio-One Cat. No. 655180) or 20 000 cells in 200 μL in μ-slide eight-well chambers (Ibidi, Gräfelfing, Germany). These seeding densities ensured confluency between ∼70 and 90% on the day of transfection. mRNA transfections were performed by removing the complete medium from the wells and replacing it with mRNA nanoparticles diluted 10 times in complete medium. Cells were exposed to mRNA nanoparticles for 2 h at 37 °C, 5% CO_2_, in a humidified incubator. For some experiments, cells were stained with 200 μL of 1 μM CellTrace yellow (Thermo Fisher Scientific, Cat. No. C34567) directly after the nanoparticle incubation, according to the manufacturer’s instructions. Regardless of the tested N/P ratio or type of nanoparticle, mRNA inputs were equalized across conditions, resulting in 107 ng/well for luciferase assays in 96-well plate formats and 214 ng/well for confocal microscopy experiments. Notably, these amounts of mRNA ensured equal doses (pg mRNA/cell) regardless of the format used for transfection. For untreated controls, complete cell culture medium was added and refreshed as often as in the experimental conditions.

### Live-Cell Confocal Laser Scanning Microscopy

Before microscopy, complete medium containing phenol red was replaced with an equal volume of phenol red-free and HEPES-formulated Opti-MEM (Gibco). The uptake of Cy5-eGFP mRNA-formulated nanoparticles was assessed 2 h post-transfection, and eGFP expression was assessed 24 h post-transfection. Live-cell imaging was performed using a Leica TCS SP8 SMD (Leica Microsystems, Mannheim, Germany) with an HC PL APO CS2 63×/1.20 water objective and a temperature-controlled stage at 36.5 °C. eGFP was excited at 488 nm (emission: 500–550 nm). CellTrace yellow was excited at 555 nm (emission: 570–620 nm), and Cy5 was excited at 633 nm (emission: 650–690 nm). All laser lines were generated by a white-light laser, and emissions were detected with hybrid detectors. All channels were sequentially acquired to avoid crosstalk at a bit depth of 12. Except for the LMM eGFP conditions (reduced gain), equal acquisition settings (pixel size, pinhole, laser power, and gain) per fluorophore were used within the experiments.

### Quantification of Fluorescence

Quantification of the Cy5 fluorescence was performed as previously described.^59,60^ In brief, five separate images (unless stated otherwise) containing approximately 10 cells per image were analyzed using ImageJ (version 1.53f51). A square mask of 184.52 μm^2^ was used to quantify the raw integrated density (sum of all pixels in a region of interest). Importantly, cell confluencies ranged from ∼80–95%, ensuring minimal impact of cell density on total fluorescence values. The brightness and contrast of the images were adjusted to find a cell-free region to determine background fluorescence. Then, a circle was drawn to measure the background fluorescence. To obtain the corrected total cellular fluorescence (CTCF), the area (in pixels) was multiplied by the mean gray value of the background and subsequently subtracted from the raw integrated density.

### Detection of Luciferase Expression

The extent of luciferase production was determined using the Nano-Glo Luciferase Assay (Promega, Madison, WI, USA, Cat No. N1130) according to the manufacturer’s instructions. Briefly, 50 μL of the sample was mixed with a 1:50 dilution of Nano-Glo luciferase assay substrate in Nano-Glo luciferase assay buffer. The resulting mixture was incubated at room temperature, hidden from light, for at least 3 min in a black, clear, flat-bottom 96-well plate (Corning Inc., Corning, NY, USA, Cat No. 3631). An intersample distance in the 96-well plate of at least two columns ensured no signal crosstalk between experimental conditions. Luminescence was measured after briefly shaking the plate using the VICTOR X3Multilabel Plate Reader (PerkinElmer, Waltham, MA, USA).

### Effect of mRNA Polyplexes on Metabolic Activity

The resazurin-based assay was performed essentially as described previously.^61,62^ In brief, the effect of different peptide-mRNA complexes on the metabolic activity of cells was measured 24 h post-transfection. Resazurin sodium salt (Sigma-Aldrich, Cat No. R7017) was dissolved in PBS and diluted 100 times to a final concentration of 100 μg/mL in complete medium. After 2 h of incubation with cells at 37 °C, fluorescence was measured using the VICTOR X3Multilabel Plate Reader (PerkinElmer, Waltham, MA, USA). After briefly shaking the plate, resazurin was excited at 485 nm, and emission was collected from 570 to 620 nm. All samples were blanked by the average signal of cell-free wells. Blanked data were normalized to untreated conditions per cell type.

### Heparin-Mediated Polyplex Decomplexation Assay

For testing the stability of mRNA polyplexes, a polyanion decomplexation assay, mediated by the polyanionic heparin (heparin sodium salt from porcine intestinal mucosa, Cat. No. H3149-100 KU, Sigma-Aldrich) was performed. mRNA polyplexes were freshly prepared with uncapped SecNLuc mRNA (RIBOPRO), as described in the section mRNA Nanoparticle Formation. Following polyplex formation, all samples were incubated for at least 30 min at room temperature. Importantly, separate stock solutions of heparin were made to ensure an equal volume across conditions regardless of heparin concentration. For decomplexation, 5 μL of 10× polyplexes were diluted in RNase-free TE-buffer at pH 7.5 (Promega, Cat. No. E260A) supplemented with an equal volume of heparin across conditions to a total volume of 50 μL in a 384-well plate (Greiner Bio-One, Cat. No. 781101). All samples were quickly spun down before incubation at 37 °C for 90 min. The QuantiFluor RNA System (Promega, Cat. No. E3310) was used with a 1:2000 dilution of RNA-binding dye for all tested conditions to detect released mRNA. For quantification, a calibration curve with uncapped SecNLuc mRNA was made, spanning from 0.19 ng mRNA/well to 50 ng mRNA/well. For detection of released mRNA, the dye was excited at 485 nm, and emission was collected at 535 nm using the VICTOR X3Multilabel Plate Reader (PerkinElmer)

### Data Analysis and Statistics

Data analysis was performed using GraphPad Prism (GraphPad Software, version 8.4.2, San Diego, CA, USA). Data are presented as means ± standard deviation (SD) unless stated otherwise. Means were calculated by first averaging the technical replicates, for which outliers were identified using Grubbs’ test, followed by averaging the biological replicates. Before statistical analysis, all data were checked for normal distribution with a Shapiro–Wilk test. Statistical analysis was done using one- or two-way analysis of variance (ANOVA). Multiple comparisons were corrected using Tukey’s test with 95% confidence intervals. For the mRNA standard curve used in heparin decomplexation assays, the coefficient of variation was determined both in the standards and in samples and was ≤20%. The standard curve was fitted using a sigmoidal four-parameter logistic curve. The standard curve was back fitted with ±10% accuracy to verify the correctness of the fit. The signals from samples were blanked with the appropriate solution (TE buffer) before calculating interpolations. *p* > 0.05 was considered not significant, and *p* values were reported using the GraphPad Prism style (*p ≤ 0.05, **p ≤ 0.01, ***p ≤ 0.001, and ****p ≤ 0.0001).

## Supporting Information Available

CPP amino acid sequences (Table S1), DLS characterization of mRNA nanoparticles (Table S2), chemical characterization of CPPs by RP-HPLC and ESI-MS (Figure S1), SDS-PAGE of ornithine- and histidine-substituted hLF CPPs (Figure S2), helical wheel projections of CPPs (Figure S3), SDS-PAGE of ornithine- and leucine-substituted hLF CPPs (Figure S4), quantification of fluorescently labeled mRNA cellular uptake (Figure S5), effect of mRNA transfections at different N/P ratios on metabolic activity (Figure S6), confocal microscopy of (Cy5-) eGFP mRNA 24 h post-transfection (Figure S7), heparin-mediated decomplexation of CPPs at different N/P ratios (Figure S8) (PDF)

## References

[ref1] Kurrikoff K.; Vunk B.; Langel D. C. Status Update in the Use of Cell-Penetrating Peptides for the Delivery of Macromolecular Therapeutics. Expert Opin. Biol. Ther. 2021, 21 (3), 361–370. 10.1080/14712598.2021.1823368. 32938243

[ref2] Lönn P.; Dowdy S. F. Cationic PTD/CPP-Mediated Macromolecular Delivery: Charging into the Cell. Expert Opin. Drug Delivery 2015, 12 (10), 1627–1636. 10.1517/17425247.2015.1046431. 25994800

[ref3] Deshayes S.; Konate K.; Dussot M.; Chavey B.; Vaissière A.; Van T. N. N.; Aldrian G.; Padari K.; Pooga M.; Vivès E.; Boisguérin P. Deciphering the Internalization Mechanism of WRAP:SiRNA Nanoparticles. Biochim. Biophys. Acta - Biomembr. 2020, 1862 (6), 183252 10.1016/j.bbamem.2020.183252. 32135145

[ref4] Konate K.; Lindberg M. F.; Vaissiere A.; Jourdan C.; Aldrian G.; Margeat E.; Deshayes S.; Boisguerin P. Optimisation of Vectorisation Property: A Comparative Study for a Secondary Amphipathic Peptide. Int. J. Pharm. 2016, 509 (1), 71–84. 10.1016/j.ijpharm.2016.05.030. 27224007

[ref5] Duchardt F.; Fotin-Mleczek M.; Schwarz H.; Fischer R.; Brock R. A Comprehensive Model for the Cellular Uptake of Cationic Cell-Penetrating Peptides. Traffic 2007, 8 (7), 848–866. 10.1111/j.1600-0854.2007.00572.x. 17587406

[ref6] Lättig-Tünnemann G.; Prinz M.; Hoffmann D.; Behlke J.; Palm-Apergi C.; Morano I.; Herce H. D.; Cardoso M. C. Backbone Rigidity and Static Presentation of Guanidinium Groups Increases Cellular Uptake of Arginine-Rich Cell-Penetrating Peptides. Nat. Commun. 2011, 2 (1), 453 10.1038/ncomms1459. 21878907 PMC3265364

[ref7] Nischan N.; Herce H. D.; Natale F.; Bohlke N.; Budisa N.; Cardoso M. C.; Hackenberger C. P. R. Covalent Attachment of Cyclic TAT Peptides to GFP Results in Protein Delivery into Live Cells with Immediate Bioavailability. Angew. Chemie Int. Ed. 2015, 54 (6), 1950–1953. 10.1002/anie.201410006. 25521313

[ref8] Verdurmen W. P. R.; Thanos M.; Ruttekolk I. R.; Gulbins E.; Brock R. Cationic Cell-Penetrating Peptides Induce Ceramide Formation via Acid Sphingomyelinase: Implications for Uptake. J. Controlled Release 2010, 147 (2), 171–179. 10.1016/j.jconrel.2010.06.030. 20620184

[ref9] Saha A.; Mandal S.; Arafiles J. V. V; Gómez-González J.; Hackenberger C. P. R.; Brik A. Structure-Uptake Relationship Study of DABCYL Derivatives Linked to Cyclic Cell-Penetrating Peptides for Live-Cell Delivery of Synthetic Proteins. Angew. Chemie Int. Ed. 2022, 61 (47), e202207551 10.1002/anie.202207551. PMC9828537 36004945

[ref10] Tong X.; Pan W.; Su T.; Zhang M.; Dong W.; Qi X. Recent Advances in Natural Polymer-Based Drug Delivery Systems. React. Funct. Polym. 2020, 148, 104501 10.1016/j.reactfunctpolym.2020.104501.

[ref11] Hajj K. A.; Whitehead K. A. Tools for Translation: Non-Viral Materials for Therapeutic MRNA Delivery. Nat. Rev. Mater. 2017, 2 (10), 17056 10.1038/natrevmats.2017.56.

[ref12] Barbier A. J.; Jiang A. Y.; Zhang P.; Wooster R.; Anderson D. G. The Clinical Progress of MRNA Vaccines and Immunotherapies. Nat. Biotechnol. 2022, 40 (6), 840–854. 10.1038/s41587-022-01294-2. 35534554

[ref13] Betts C.; Saleh A. F.; Arzumanov A. A.; Hammond S. M.; Godfrey C.; Coursindel T.; Gait M. J.; Wood M. J. A. Pip6-PMO, A New Generation of Peptide-Oligonucleotide Conjugates With Improved Cardiac Exon Skipping Activity for DMD Treatment. Mol. Ther. - Nucleic Acids 2012, 1, e38 10.1038/mtna.2012.30. 23344180 PMC3438601

[ref14] Brock R. The Uptake of Arginine-Rich Cell-Penetrating Peptides: Putting the Puzzle Together. Bioconjugate Chem. 2014, 25 (5), 863–868. 10.1021/bc500017t. 24679171

[ref15] Ezzat K.; Helmfors H.; Tudoran O.; Juks C.; Lindberg S.; Padari K.; El-Andaloussi S.; Pooga M.; Langel U. Scavenger Receptor-Mediated Uptake of Cell-Penetrating Peptide Nanocomplexes with Oligonucleotides. FASEB J. Off. Publ. Fed. Am. Soc. Exp. Biol. 2012, 26 (3), 1172–1180. 10.1096/fj.11-191536. 22138034

[ref16] van Asbeck A. H.; Beyerle A.; McNeill H.; Bovee-Geurts P. H. M.; Lindberg S.; Verdurmen W. P. R.; Hällbrink M.; Langel D. C.; Heidenreich O.; Brock R. Molecular Parameters of SiRNA-Cell Penetrating Peptide Nanocomplexes for Efficient Cellular Delivery. ACS Nano 2013, 7 (5), 3797–3807. 10.1021/nn305754c. 23600610

[ref17] Ezzat K.; EL Andaloussi S.; Zaghloul E. M.; Lehto T.; Lindberg S.; Moreno P. M. D.; Viola J. R.; Magdy T.; Abdo R.; Guterstam P.; Sillard R.; Hammond S. M.; Wood M. J. A.; Arzumanov A. A.; Gait M. J.; Smith C. I. E.; Hallbrink M.; Langel U. PepFect 14, a Novel Cell-Penetrating Peptide for Oligonucleotide Delivery in Solution and as Solid Formulation. Nucleic Acids Res. 2011, 39 (12), 5284–5298. 10.1093/nar/gkr072. 21345932 PMC3130259

[ref18] Veiman K.-L.; Mäger I.; Ezzat K.; Margus H.; Lehto T.; Langel K.; Kurrikoff K.; Arukuusk P.; Suhorutšenko J.; Padari K.; Pooga M.; Lehto T.; Langel D. C. PepFect14 Peptide Vector for Efficient Gene Delivery in Cell Cultures. Mol. Pharmaceutics 2013, 10 (1), 199–210. 10.1021/mp3003557. 23186360

[ref19] Van Der Bent M. L.; Filho O. P.; Willemse M.; Hallbrink M.; Wansink D. G.; Brock R. The Nuclear Concentration Required for Antisense Oligonucleotide Activity in Myotonic Dystrophy Cells. FASEB J. 2019, 33 (10), 11314–11325. 10.1096/fj.201900263R. 31311315

[ref20] van den Brand D.; Gorris M. A. J.; van Asbeck A. H.; Palmen E.; Ebisch I.; Dolstra H.; Hällbrink M.; Massuger L. F. A. G.; Brock R. Peptide-Mediated Delivery of Therapeutic MRNA in Ovarian Cancer. Eur. J. Pharm. Biopharm. Off. J. Arbeitsgemeinschaft fur Pharm. Verfahrenstechnik e.V 2019, 141, 180–190. 10.1016/j.ejpb.2019.05.014. 31103743

[ref21] Luna Velez M. V.; Paulino da Silva Filho O.; Verhaegh G. W.; van Hooij O.; El Boujnouni N.; Brock R.; Schalken J. A. Delivery of Antisense Oligonucleotides for Splice-Correction of Androgen Receptor Pre-MRNA in Castration-Resistant Prostate Cancer Models Using Cell-Penetrating Peptides. Prostate 2022, 82 (6), 657–665. 10.1002/pros.24309. 35098567 PMC9303360

[ref22] Yandek L. E.; Pokorny A.; Florén A.; Knoelke K.; Langel U.; Almeida P. F. F. Mechanism of the Cell-Penetrating Peptide Transportan 10 Permeation of Lipid Bilayers. Biophys. J. 2007, 92 (7), 2434–2444. 10.1529/biophysj.106.100198. 17218466 PMC1864827

[ref23] Mäe M.; El Andaloussi S.; Lundin P.; Oskolkov N.; Johansson H. J.; Guterstam P.; Langel U. A Stearylated CPP for Delivery of Splice Correcting Oligonucleotides Using a Non-Covalent Co-Incubation Strategy. J. Control. release Off. J. Control. Release Soc. 2009, 134 (3), 221–227. 10.1016/j.jconrel.2008.11.025. 19105971

[ref24] Ramsay E.; Gumbleton M. Polylysine and Polyornithine Gene Transfer Complexes: A Study of Complex Stability and Cellular Uptake as a Basis for Their Differential in-Vitro Transfection Efficiency. J. Drug Target. 2002, 10 (1), 1–9. 10.1080/10611860290007487. 11996081

[ref25] Falato L.; Gestin M.; Langel D. C. PepFect14 Signaling and Transfection. Methods Mol. Biol. 2022, 2383, 229–246. 10.1007/978-1-0716-1752-6_15. 34766293

[ref26] Midoux P.; Pichon C.; Yaouanc J.-J.; Jaffrès P.-A. Chemical Vectors for Gene Delivery: A Current Review on Polymers, Peptides and Lipids Containing Histidine or Imidazole as Nucleic Acids Carriers. Br. J. Pharmacol. 2009, 157 (2), 166–178. 10.1111/j.1476-5381.2009.00288.x. 19459843 PMC2697805

[ref27] Won Y.-W.; Yoon S.-M.; Lee K.-M.; Kim Y.-H. Poly(Oligo-D-Arginine) With Internal Disulfide Linkages as a Cytoplasm-Sensitive Carrier for SiRNA Delivery. Mol. Ther. 2011, 19 (2), 372–380. 10.1038/mt.2010.242. 21081902 PMC3034849

[ref28] Kiselev A.; Egorova A.; Laukkanen A.; Baranov V.; Urtti A. Characterization of Reducible Peptide Oligomers as Carriers for Gene Delivery. Int. J. Pharm. 2013, 441 (1), 736–747. 10.1016/j.ijpharm.2012.10.020. 23089582

[ref29] Godbey W. T.; Wu K. K.; Mikos A. G. Size Matters: Molecular Weight Affects the Efficiency of Poly(Ethylenimine) as a Gene Delivery Vehicle. J. Biomed. Mater. Res. 1999, 45 (3), 268–275. 10.1002/(SICI)1097-4636(19990605)45:3<268::AID-JBM15>3.0.CO;2-Q. 10397985

[ref30] Lin C.; Zhong Z.; Lok M. C.; Jiang X.; Hennink W. E.; Feijen J.; Engbersen J. F. J. Novel Bioreducible Poly(Amido Amine)s for Highly Efficient Gene Delivery. Bioconjugate Chem. 2007, 18 (1), 138–145. 10.1021/bc060200l. 17226966

[ref31] Zhao X.; Glass Z.; Chen J.; Yang L.; Kaplan D. L.; Xu Q. MRNA Delivery Using Bioreducible Lipidoid Nanoparticles Facilitates Neural Differentiation of Human Mesenchymal Stem Cells. Adv. Healthc. Mater. 2021, 10 (4), 2000938 10.1002/adhm.202000938. 32815325

[ref32] Krhač Levačić A.; Berger S.; Müller J.; Wegner A.; Lächelt U.; Dohmen C.; Rudolph C.; Wagner E. Dynamic MRNA Polyplexes Benefit from Bioreducible Cleavage Sites for in Vitro and in Vivo Transfer. J. Control. release Off. J. Control. Release Soc. 2021, 339, 27–40. 10.1016/j.jconrel.2021.09.016. 34547258

[ref33] Hickey J. C.; Hurst P. J.; Patterson J. P.; Guan Z. Facile Synthesis of Multifunctional Bioreducible Polymers for MRNA Delivery. Chemistry 2023, 29 (12), e202203393 10.1002/chem.202203393. 36469740

[ref34] Bulmus V.; Woodward M.; Lin L.; Murthy N.; Stayton P.; Hoffman A. A New PH-Responsive and Glutathione-Reactive, Endosomal Membrane-Disruptive Polymeric Carrier for Intracellular Delivery of Biomolecular Drugs. J. Control. release Off. J. Control. Release Soc. 2003, 93 (2), 105–120. 10.1016/j.jconrel.2003.06.001. 14636717

[ref35] Ou M.; Wang X.-L.; Xu R.; Chang C.-W.; Bull D. A.; Kim S. W. Novel Biodegradable Poly(Disulfide Amine)s for Gene Delivery with High Efficiency and Low Cytotoxicity. Bioconjugate Chem. 2008, 19 (3), 626–633. 10.1021/bc700397x. PMC2754712 18314939

[ref36] Gao W.; Li T.; Wang J.; Zhao Y.; Wu C. Thioether-Bonded Fluorescent Probes for Deciphering Thiol-Mediated Exchange Reactions on the Cell Surface. Anal. Chem. 2017, 89 (1), 937–944. 10.1021/acs.analchem.6b04096. 27976862

[ref37] Duchardt F.; Ruttekolk I. R.; Verdurmen W. P. R.; Lortat-Jacob H.; Burck J.; Hufnagel H.; Fischer R.; van den Heuvel M.; Lowik D. W. P. M.; Vuister G. W.; Ulrich A.; de Waard M.; Brock R. A Cell-Penetrating Peptide Derived from Human Lactoferrin with Conformation-Dependent Uptake Efficiency. J. Biol. Chem. 2009, 284 (52), 36099–36108. 10.1074/jbc.M109.036426. 19858187 PMC2794725

[ref38] Wallbrecher R.; Verdurmen W. P. R.; Schmidt S.; Bovee-Geurts P. H.; Broecker F.; Reinhardt A.; van Kuppevelt T. H.; Seeberger P. H.; Brock R. The Stoichiometry of Peptide-Heparan Sulfate Binding as a Determinant of Uptake Efficiency of Cell-Penetrating Peptides. Cell. Mol. Life Sci. 2013, 71 (14), 2717–2729. 10.1007/s00018-013-1517-8. 24270856 PMC11113137

[ref39] van Asbeck A. H.; Dieker J.; Oude Egberink R.; van den Berg L.; van der Vlag J.; Brock R. Protein Expression Correlates Linearly with MRNA Dose over Up to Five Orders of Magnitude In Vitro and In Vivo. Biomedicines. 2021, 9, 511 10.3390/biomedicines9050511. 34063094 PMC8148180

[ref40] Oude Egberink R.; Zegelaar H. M.; El Boujnouni N.; Versteeg E. M. M.; Daamen W. F.; Brock R. Biomaterial-Mediated Protein Expression Induced by Peptide-MRNA Nanoparticles Embedded in Lyophilized Collagen Scaffolds. Pharmaceutics. 2022, 14, 1619 10.3390/pharmaceutics14081619. 36015245 PMC9414905

[ref41] Palacio-Castañeda V.; Oude Egberink R.; Sait A.; Andrée L.; Sala B. M.; Hassani Besheli N.; Oosterwijk E.; Nilvebrant J.; Leeuwenburgh S. C. G.; Brock R.; Verdurmen W. P. R. Mimicking the Biology of Engineered Protein and MRNA Nanoparticle Delivery Using a Versatile Microfluidic Platform. Pharmaceutics. 2021, 13, 1944 10.3390/pharmaceutics13111944. 34834361 PMC8624409

[ref42] Bresson S.; Tollervey D. Surveillance-Ready Transcription: Nuclear RNA Decay as a Default Fate. Open Biol. 2018, 8 ( (3), ), 10.1098/rsob.170270. PMC5881035 29563193

[ref43] Chuard N.; Gasparini G.; Roux A.; Sakai N.; Matile S. Cell-Penetrating Poly(Disulfide)s: The Dependence of Activity, Depolymerization Kinetics and Intracellular Localization on Their Length. Org. Biomol. Chem. 2015, 13 (1), 64–67. 10.1039/C4OB02060J. 25375762

[ref44] Smithies O. Disulfide-Bond Cleavage and Formation in Proteins. Science (80-.). 1965, 150 (3703), 1595–1598. 10.1126/science.150.3703.1595. 5866656

[ref45] Monahan F. J.; German J. B.; Kinsella J. E. Effect of PH and Temperature on Protein Unfolding and Thiol/Disulfide Interchange Reactions during Heat-Induced Gelation of Whey Proteins. J. Agric. Food Chem. 1995, 43 (1), 46–52. 10.1021/jf00049a010.

[ref46] Hopp T. P.; Woods K. R. A Computer Program for Predicting Protein Antigenic Determinants. Mol. Immunol. 1983, 20 (4), 483–489. 10.1016/0161-5890(83)90029-9. 6191210

[ref47] Andaloussi S. E.; Guterstam P.; Langel U. Assessing the Delivery Efficacy and Internalization Route of Cell-Penetrating Peptides. Nat. Protoc. 2007, 2 (8), 2043–2047. 10.1038/nprot.2007.302. 17703217

[ref48] Favretto M. E.; Wallbrecher R.; Schmidt S.; van de Putte R.; Brock R. Glycosaminoglycans in the Cellular Uptake of Drug Delivery Vectors - Bystanders or Active Players?. J. Controlled Release 2014, 180, 81–90. 10.1016/j.jconrel.2014.02.011. 24548480

[ref49] Lo S. L.; Wang S. An Endosomolytic Tat Peptide Produced by Incorporation of Histidine and Cysteine Residues as a Nonviral Vector for DNA Transfection. Biomaterials 2008, 29 (15), 2408–2414. 10.1016/j.biomaterials.2008.01.031. 18295328

[ref50] Tanaka K.; Kanazawa T.; Ogawa T.; Takashima Y.; Fukuda T.; Okada H. Disulfide Crosslinked Stearoyl Carrier Peptides Containing Arginine and Histidine Enhance SiRNA Uptake and Gene Silencing. Int. J. Pharm. 2010, 398 (1), 219–224. 10.1016/j.ijpharm.2010.07.038. 20674725

[ref51] Lointier M.; Dussouillez C.; Glattard E.; Kichler A.; Bechinger B. Different Biological Activities of Histidine-Rich Peptides Are Favored by Variations in Their Design. Toxins. 2021, 13, 363 10.3390/toxins13050363. 34065185 PMC8160934

[ref52] Mason A. J.; Leborgne C.; Moulay G.; Martinez A.; Danos O.; Bechinger B.; Kichler A. Optimising Histidine Rich Peptides for Efficient DNA Delivery in the Presence of Serum. J. Controlled Release 2007, 118 (1), 95–104. 10.1016/j.jconrel.2006.12.004. 17254661

[ref53] Soomets U.; Lindgren M.; Gallet X.; Hällbrink M.; Elmquist A.; Balaspiri L.; Zorko M.; Pooga M.; Brasseur R.; Langel D. C. Deletion Analogues of Transportan. Biochim. Biophys. Acta - Biomembr. 2000, 1467 (1), 165–176. 10.1016/S0005-2736(00)00216-9. 10930519

[ref54] Feiner-Gracia N.; Olea R. A.; Fitzner R.; El Boujnouni N.; van Asbeck A. H.; Brock R.; Albertazzi L. Super-Resolution Imaging of Structure, Molecular Composition, and Stability of Single Oligonucleotide Polyplexes. Nano Lett. 2019, 19 (5), 2784–2792. 10.1021/acs.nanolett.8b04407. 31001985 PMC6509642

[ref55] Hou X.; Zaks T.; Langer R.; Dong Y. Lipid Nanoparticles for MRNA Delivery. Nat. Rev. Mater. 2021, 6 (12), 1078–1094. 10.1038/s41578-021-00358-0. 34394960 PMC8353930

[ref56] Grimme C. J.; Hanson M. G.; Reineke T. M. Enhanced ASO-Mediated Gene Silencing with Lipophilic PH-Responsive Micelles. Bioconjugate Chem. 2023, 34 (7), 1244–1257. 10.1021/acs.bioconjchem.3c00133. 37384839

[ref57] Hanson M. G.; Grimme C. J.; Santa Chalarca C. F.; Reineke T. M. Cationic Micelles Outperform Linear Polymers for Delivery of Antisense Oligonucleotides in Serum: An Exploration of Polymer Architecture, Cationic Moieties, and Cell Addition Order. Bioconjugate Chem. 2022, 33 (11), 2121–2131. 10.1021/acs.bioconjchem.2c00379. 36265078

[ref58] Schlich M.; Palomba R.; Costabile G.; Mizrahy S.; Pannuzzo M.; Peer D.; Decuzzi P. Cytosolic Delivery of Nucleic Acids: The Case of Ionizable Lipid Nanoparticles. Bioeng. Transl. Med. 2021, 6 (2), e10213 10.1002/btm2.10213. 33786376 PMC7995196

[ref59] McCloy R. A.; Rogers S.; Caldon C. E.; Lorca T.; Castro A.; Burgess A. Partial Inhibition of Cdk1 in G2 Phase Overrides the SAC and Decouples Mitotic Events. Cell Cycle 2014, 13 (9), 1400–1412. 10.4161/cc.28401. 24626186 PMC4050138

[ref60] Favretto M. E.; Brock R. Stereoselective Uptake of Cell-Penetrating Peptides Is Conserved in Antisense Oligonucleotide Polyplexes. Small 2015, 11 (12), 1414–1417. 10.1002/smll.201402101. 25382156

[ref61] van den Brand D.; van Lith S. A. M.; de Jong J. M.; Gorris M. A. J.; Palacio-Castañeda V.; Couwenbergh S. T.; Goldman M. R. G.; Ebisch I.; Massuger L. F.; Leenders W. P. J.; Brock R.; Verdurmen W. P. R. EpCAM-Binding DARPins for Targeted Photodynamic Therapy of Ovarian Cancer. Cancers. 2020, 12, 1762 10.3390/cancers12071762. 32630661 PMC7409335

[ref62] Palacio-Castañeda V.; Dumas S.; Albrecht P.; Wijgers T. J.; Descroix S.; Verdurmen W. P. R. A Hybrid In Silico and Tumor-on-a-Chip Approach to Model Targeted Protein Behavior in 3D Microenvironments. Cancers. 2021, 13, 2461 10.3390/cancers13102461. 34070171 PMC8158470

