# Supplementary material for: Deciphering Structural Determinants Distinguishing Active from Inactive Cell-Penetrating Peptides for Cytosolic mRNA Delivery
Source: Bioconjug Chem. 2023 Sep 21;34(10):1822–34. doi: 10.1021/acs.bioconjchem.3c00346 (PMC10587869; doi:10.1021/acs.bioconjchem.3c00346)
Supplement: Supplementary file 1 — bc3c00346_si_001.pdf [file bc3c00346_si_001.pdf]

Supporting Information for

Deciphering Structural Determinants Distinguishing Active from Inactive Cell-Penetrating Peptides for Cytosolic mRNA Delivery

Rik Oude Egberink[1,#], Alexander H. van Asbeck[1,#,$], Milou Boswinkel[1], Grigor Muradjan[1], Jürgen Dieker[1,&], Roland Brock[1,2,*]

[1] Department of Medical BioSciences, Research Institute for Medical Innovation, Radboud University Medical Center, 6525 GA Nijmegen, the Netherlands.
[2]Department of Medical Biochemistry, College of Medicine and Medical Sciences, Arabian Gulf University, Manama 329, Bahrain
[#] These authors contributed equally

*Corresponding author:
Prof. Dr. Roland Brock, Dept. of Medical BioSciences , Radboud University Medical Center, Geert Grooteplein 28, 6525 GA Nijmegen, The Netherlands, roland.brock@radboudumc.nl

[$]present address:
RIBOPRO B. V.
Pivot Park,
Kloosterstraat 9
5349 AB Oss, The Netherlands

[$]present address:
Mercurna B. V.
Pivot Park,
Kloosterstraat 9
5349 AB Oss, The Netherlands

Table S1: Peptide sequences and molecular weights. Cationic residues are highlighted in bold, and underlines represent amphipathic residues from PF14 that were transferred to hLF variants. The letter O denotes the non-proteinogenic amino acid ornithine. hLF: human lactoferrin-derived peptide. MW: Molecular weight.

Name	Amino Acid Sequence	Predicted MW (g/mol)	Measured MW (g/mol)	N-terminus	C-terminus
PepFect14	AGYLLG**K**LL**OO**LAAAAL**OO**LL	2406	2406	Stearylated ($C_{18}H_{38}O$)	Amidated ($CONH_2$)
hLF-WT	**K**CFQWQ**R**NM**RK**V**R**GPPVSCI**KR**	2759	2759	Acylated (CH_3CO)	
hLF-Orn	**O**CFQWQ**O**NM**OO**V**O**GPPVSCI**OO**	2549	2550		
hLF-His	**H**CFQWQ**H**NM**HH**V**H**GPPVSCI**HH**	2710	2710		
hLF-His&Orn	**O**CFQWQ**H**NM**OH**V**H**GPPVSCI**OH**	2641	2641		
hLF-v1	**K**CFQWL**R**NL**RK**V**R**ALLVSCL**KR**	2730	2731	None (H)	
hLF-v2	**K**CFQWL**K**NL**KK**V**K**ALLVSCL**KK**	2618	2619		
hLF-v3	**O**CFQWL**O**NL**OO**V**O**ALLVSCL**OO**	2520	2521		

Table S2: Characterization of nanoparticles by Dynamic Light Scattering. Averages and standard deviations reflect three or four repeated measurements. PdI: polydispersity index.

Peptide	Concentration (μM)	N/P	mRNA	Z-Average (Ø nm)	PdI	Attenuator	Intercept	Derived Counts
PF14	42	3	SecNLuc	47.9 ± 1.18	0.304 ± 0.035	10	0.918 ± 0.005	1,529 ± 41
	70	5		55.4 ± 3.69	0.402 ± 0.029	10	0.797 ± 0.043	1,886 ± 73
Mono-hLF-WT	50	3	SecNLuc	115 ± 1.03	0.008 ± 0.005	6	0.949 ± 0.002	77,646 ± 514
		5		124 ± 1.26	0.184 ± 0.009	7	0.933 ± 0.002	33,444 ± 449
Poly-hLF-WT	50	3	SecNLuc	43.4 ± 0.38	0.137 ± 0.010	9	0.932 ± 0.003	3,056 ± 16
		5		53.2 ± 1.72	0.366 ± 0.018	10	0.942 ± 0.003	1,662 ± 44
Mono-hLF-Orn	30	5	eGFP	49.4 ± 0.19	0.078 ± 0.005	9	0.936 ± 0.007	3,079 ± 31
Poly-hLF-Orn	30	5	Cy5-eGFP	72.1 ± 1.73	0.213 ± 0.058	7	0.260 ± 0.012	19,920 ± 594
Poly-hLF-Orn	30	5	eGFP	176 ± 0.01	0.027 ± 0.003	6	0.957 ± 0.003	58,679 ± 483
Mono-hLF-His	34	3	SecNLuc	198 ± 1.78	0.037 ± 0.024	7	0.937 ± 0.002	25,708 ± 295
Poly-hLF-His	50	3	SecNLuc	133 ± 1.12	0.090 ± 0.014	7	0.945 ± 0.001	20,510 ± 159
Mono-hLF-His&Orn	82.5	3	SecNLuc	127 ± 0.24	0.047 ± 0.021	6	0.934 ± 0.004	123,343 ± 506
Poly-hLF-His&Orn	82.5	3	SecNLuc	192 ± 15.6	0.038 ± 0.035	6	0.935 ± 0.001	96,156 ± 695
	70	5	eGFP	158 ± 0.85	0.037 ± 0.012	6	0.945 ± 0.001	75,300 ± 323
Mono-hLF-v1	20	3	Cy5-eGFP	198 ± 35.0	0.446 ± 0.022	7	0.249 ± 0.018	28,290 ± 371
Poly-hLF-v1	20	3	Cy5-eGFP	72.2 ± 2.11	0.214 ± 0.072	7	0.245 ± 0.021	27,984 ± 574
Mono-hLF-v2	20	3	Cy5-eGFP	182 ± 1.93	0.271 ± 0.032	7	0.293 ± 0.009	21,852 ± 177
Poly-hLF-v2	20	3	Cy5-eGFP	74.1 ± 5.78	0.343 ± 0.069	7	0.295 ± 0.383	22,749 ± 136
Mono-hLF-v3	20	3	Cy5-eGFP	109 ± 9.26	0.267 ± 0.021	7	0.241 ± 0.007	27,984 ± 573
Poly-hLF-v3	20	3	Cy5-eGFP	87.8 ± 13.8	0.312 ± 0.061	7	0.049 ± 0.001	23,313 ± 447
LMM	NA	NA	SecNLuc	570 ± 28.0	0.322 ± 0.043	10	0.886 ± 0.016	842 ± 33

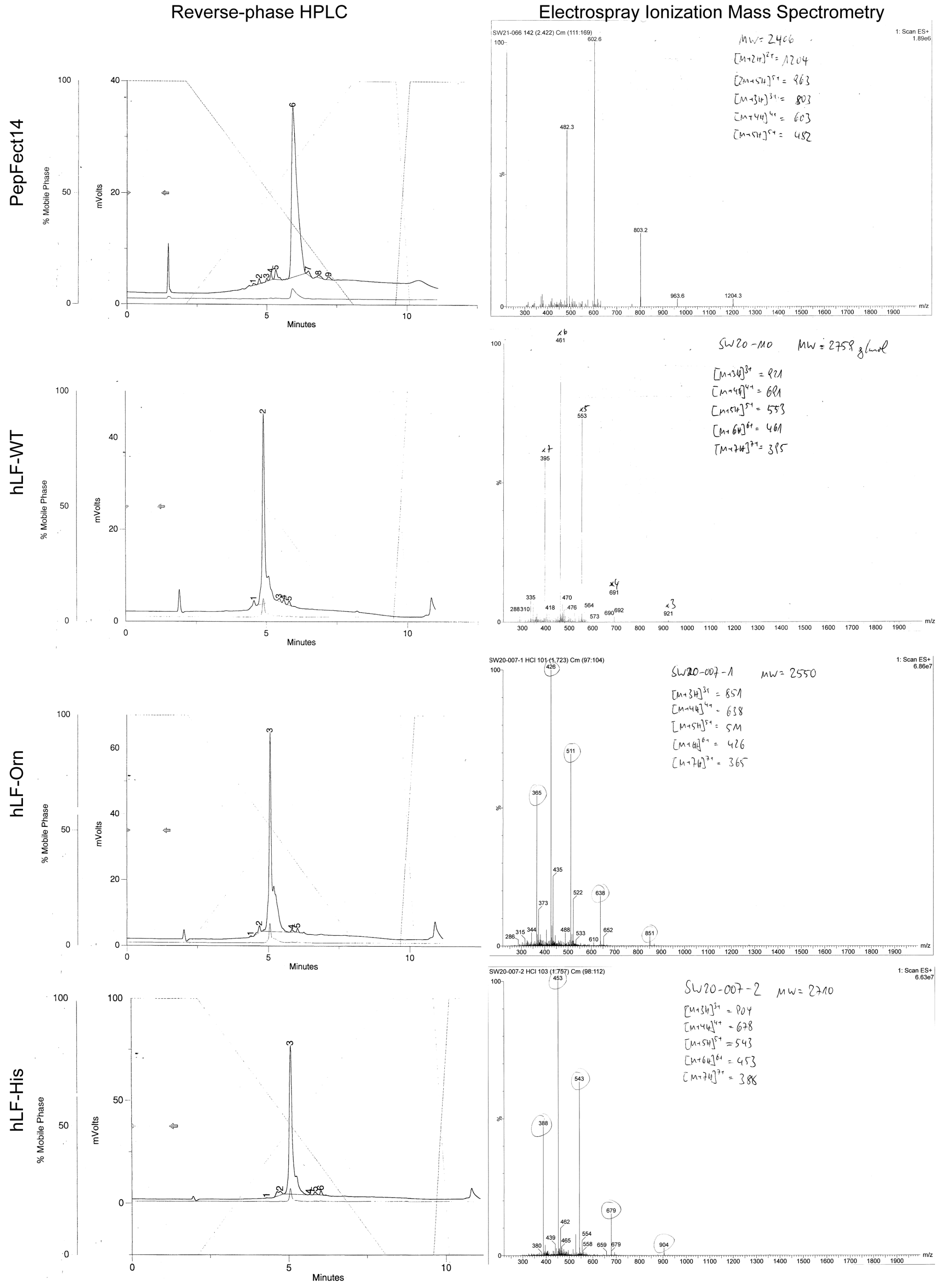
Reverse-phase HPLC
Electrospray Ionization Mass Spectrometry
PepFect14
hLF-WT
hLF-Orn
hLF-His
% Mobile Phase
mVolts
Minutes
m/z
SW21-066 142 (2.422) Cm (111:169)
1: Scan ES+
1.89e6
MW = 2406
SW20-110 MW = 2758 g/mol
SW20-007-1 HCl 101 (1.723) Cm (97:104)
1: Scan ES+
6.86e7
SW20-007-1 MW = 2550
SW20-007-2 HCl 103 (1.757) Cm (98:112)
1: Scan ES+
6.63e7
SW20-007-2 MW = 2710

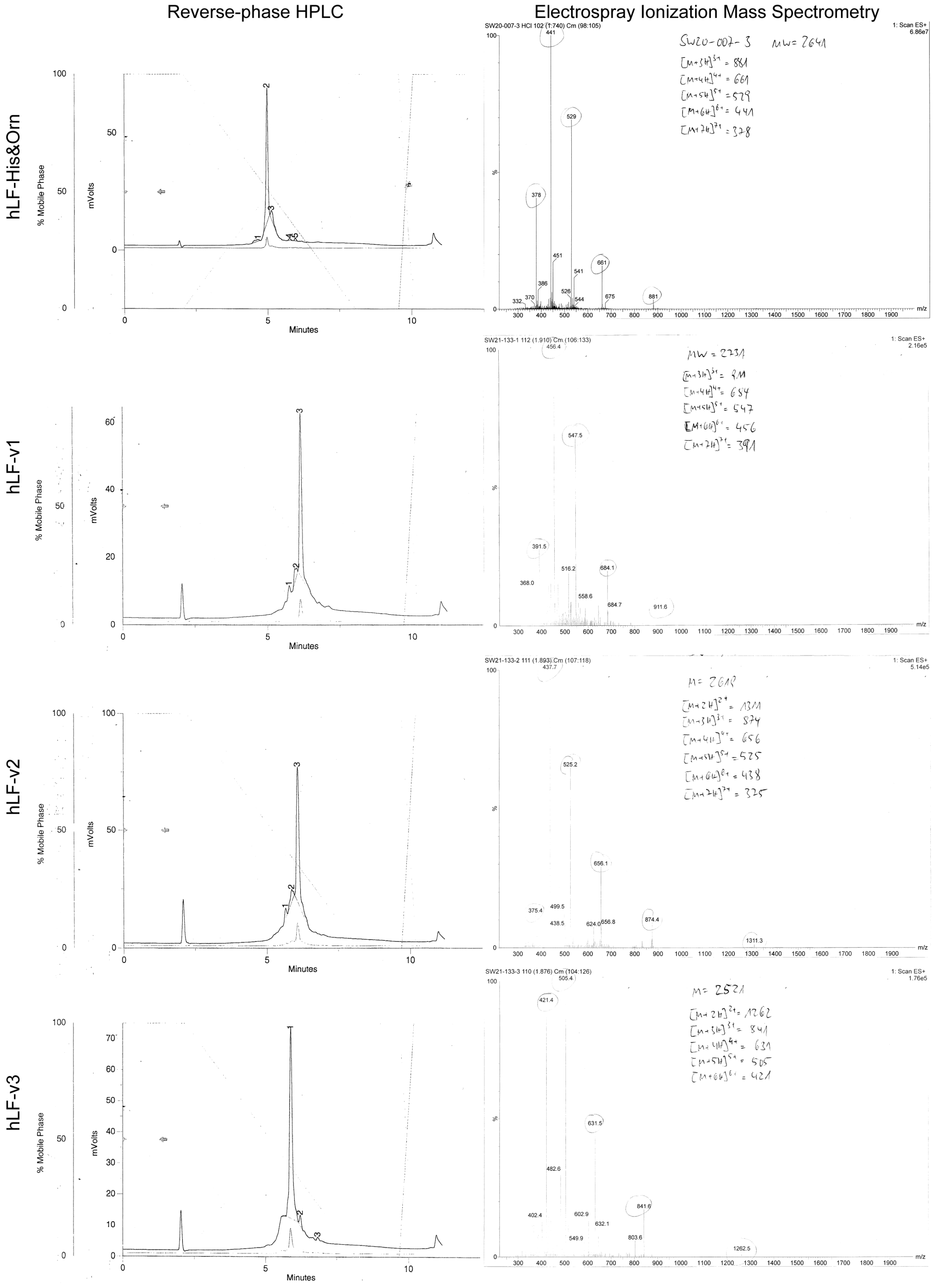

Figure S1: Chemical characterization of cell-penetrating peptides.

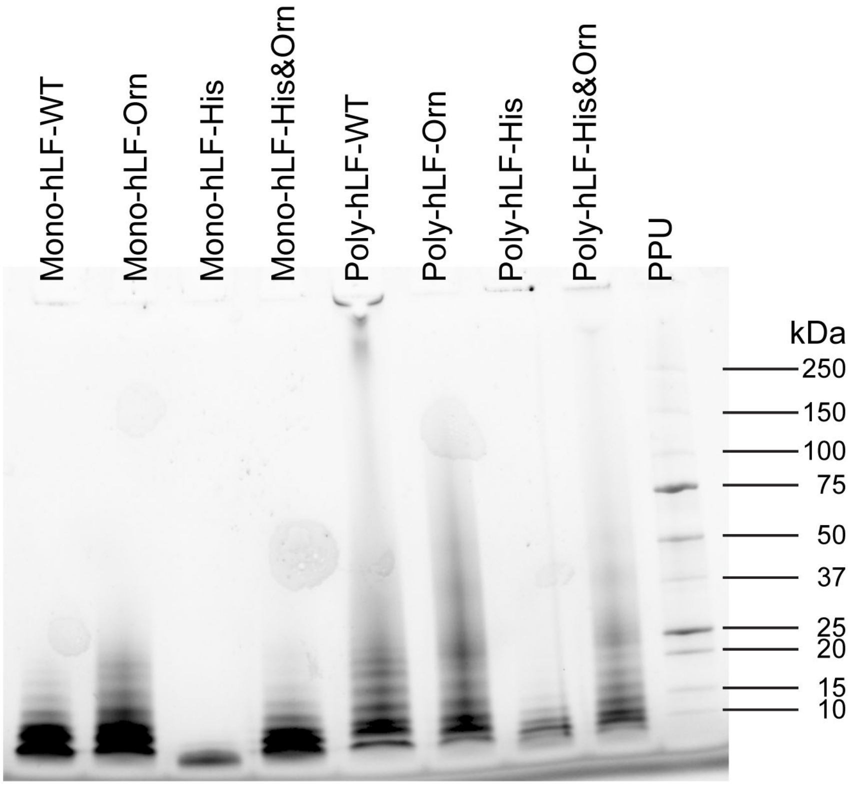

Figure S2: Oligomerization of ornithine and histidine-substituted hLF peptide variants into higher-order oligomers. For mono-hLF-x samples, peptides were incubated at a concentration of 5 mM for 2 h at 37 °C, and for the poly-hLF-x samples, peptides were incubated at a concentration of 50 mM for 24 h at 37 °C. Protein concentrations are equalized across conditions, and samples were run on a stain-free gel for direct visualization. PPU: Precision Plus Protein Unstained Protein Standards.

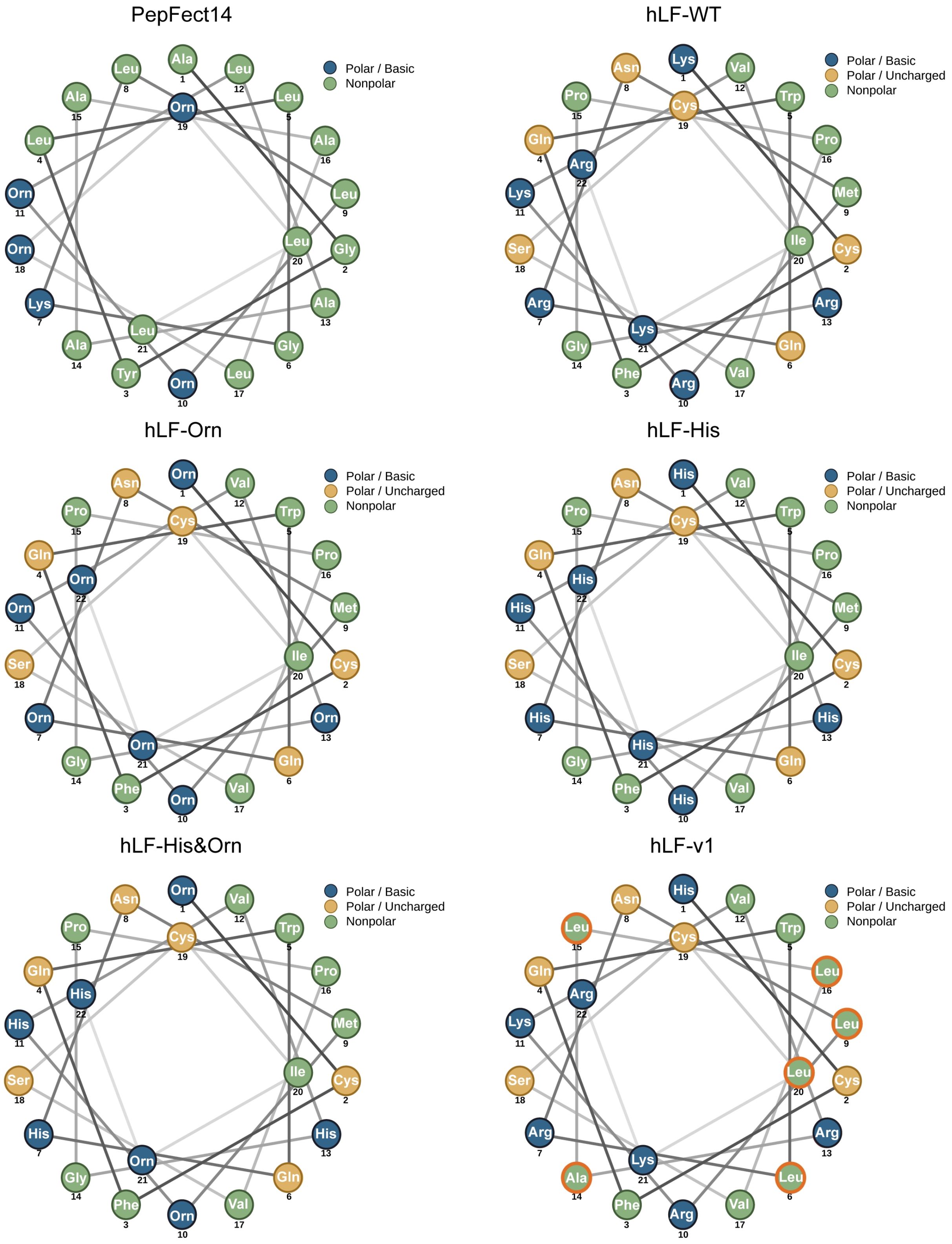

Figure S3: Helical wheel projections of PF14 and hLF variants. Numbers indicate residues from N- to C-terminus, Orn denotes ornithine. Amphipathic PF14 structural motifs incorporated into hLF-v1 are delineated in orange. Projections were made with the NetWheels application[1].

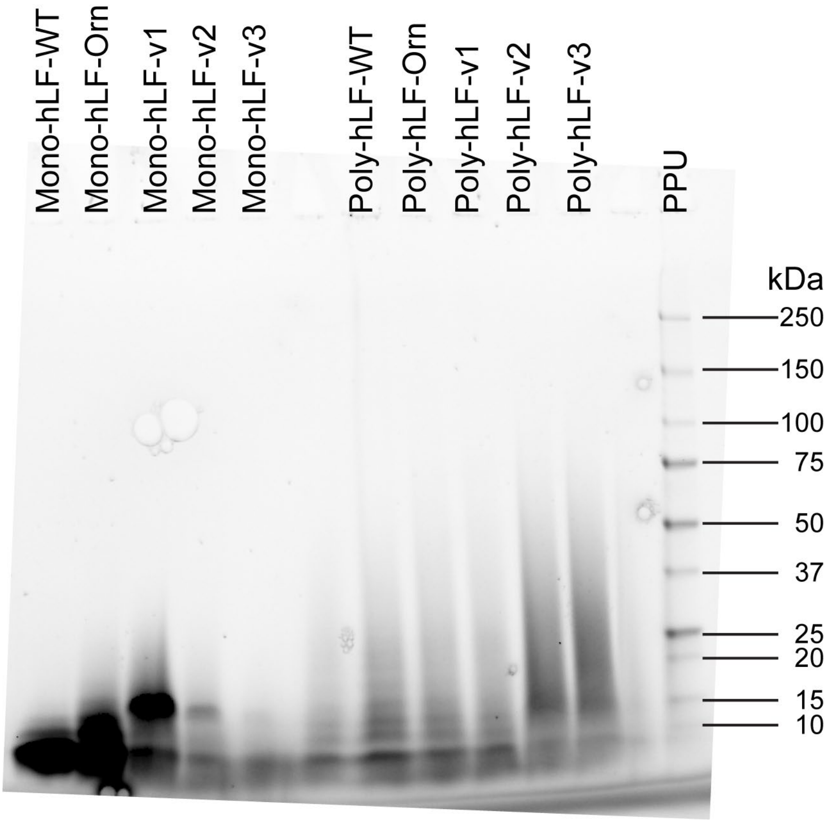

Figure S4: Oligomerization of ornithine and leucine-substituted hLF peptide variants into higher-order oligomers. For mono-hLF-x samples, peptides were incubated at a concentration of 5 mM for 2 h at 37 °C, and for the poly-hLF-x samples, peptides were incubated at a concentration of 50 mM for 24 h at 37 °C. Protein concentrations are equalized across conditions, and samples were run on a stain-free gel for direct visualization. PPU: Precision Plus Protein Unstained Protein Standards.

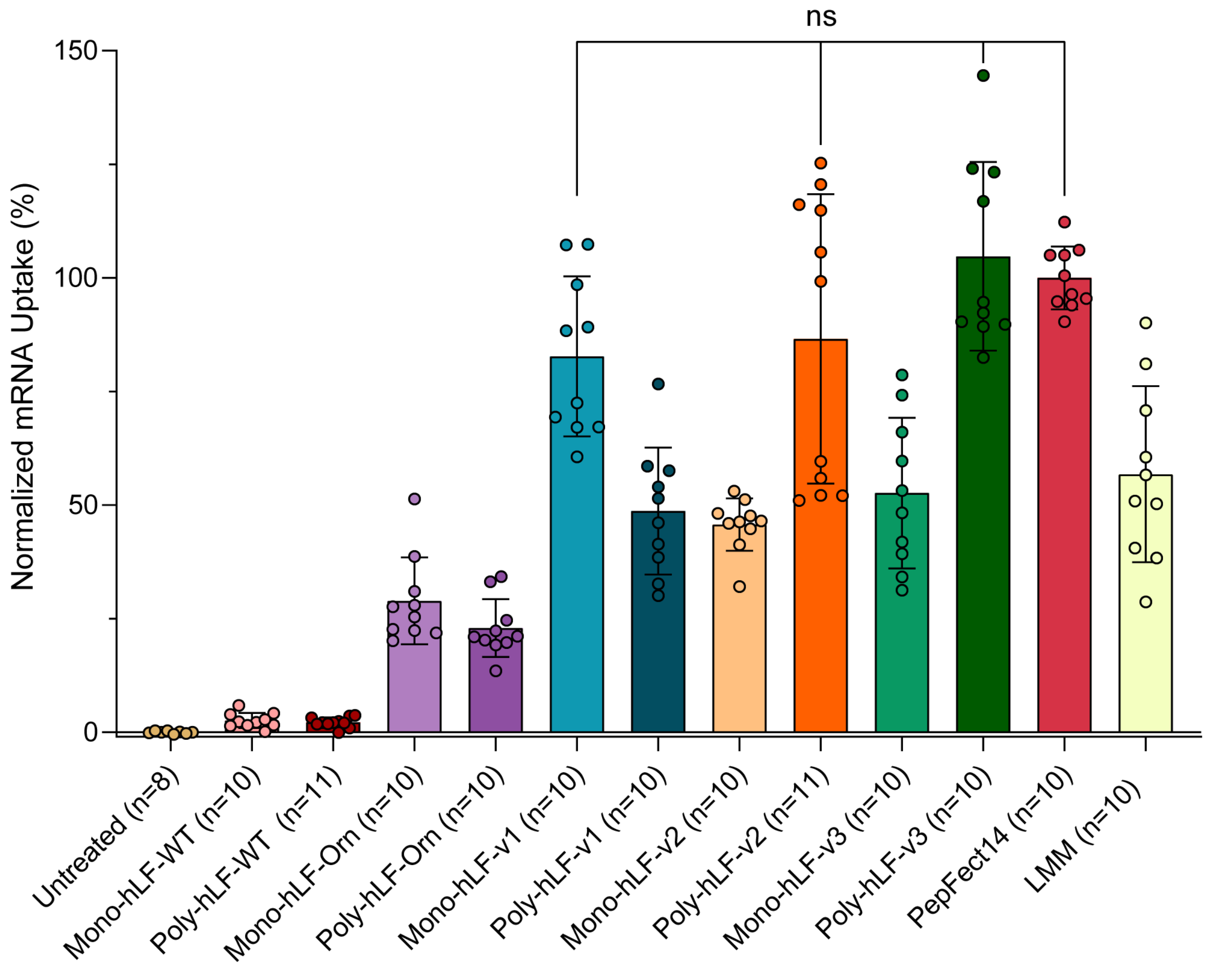

Figure S5: The effect of incorporating amphipathic structural motives of PF14 into the hLF peptide backbone on mRNA uptake efficiencies. Quantification of Cy5-eGFP mRNA uptake 2 h post-transfection of approximately 10 full fields of view per condition. Data are the normalized averages of four independent experiments and represents the mean ± SD. Data were normalized by setting the calculated corrected total cellular fluorescence (CTCF) of the untreated control to 0% and the CTCF of PepFect14 to 100% per experiment. All conditions were compared to PF14, and only non-significant differences are depicted.

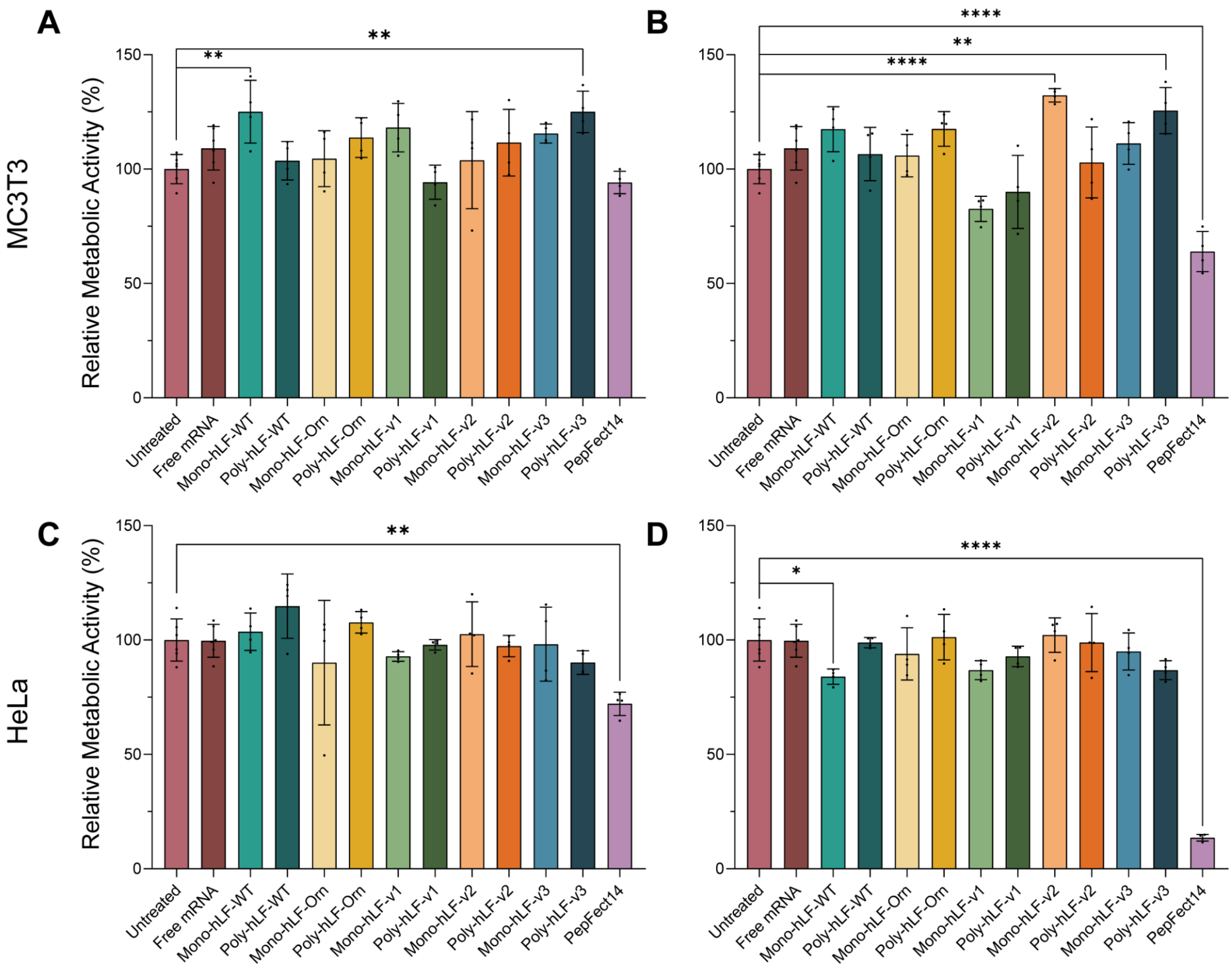

Figure S6. Effect on the metabolic activity of monomeric and polymeric hLF variants as measured by resazurin assay at nitrogen over phosphate ratios (N/P) of 3 or 5. (**A**) Effect of mRNA transfection on the metabolic activity of MC3T3 cells, as measured by resazurin assay at N/P 3 (**B**) and N/P 5. (**C**) Effect of mRNA transfection on the metabolic activity of HeLa cells, as measured by resazurin assay at N/P 3 (**D**) and N/P 5. Resazurin data was normalized to the untreated condition. . All conditions were compared to untreated, and only significant differences are depicted. * $p \leq 0.05$, ** $p \leq 0.01$, *** $p \leq 0.001$, and **** $p \leq 0.0001$.

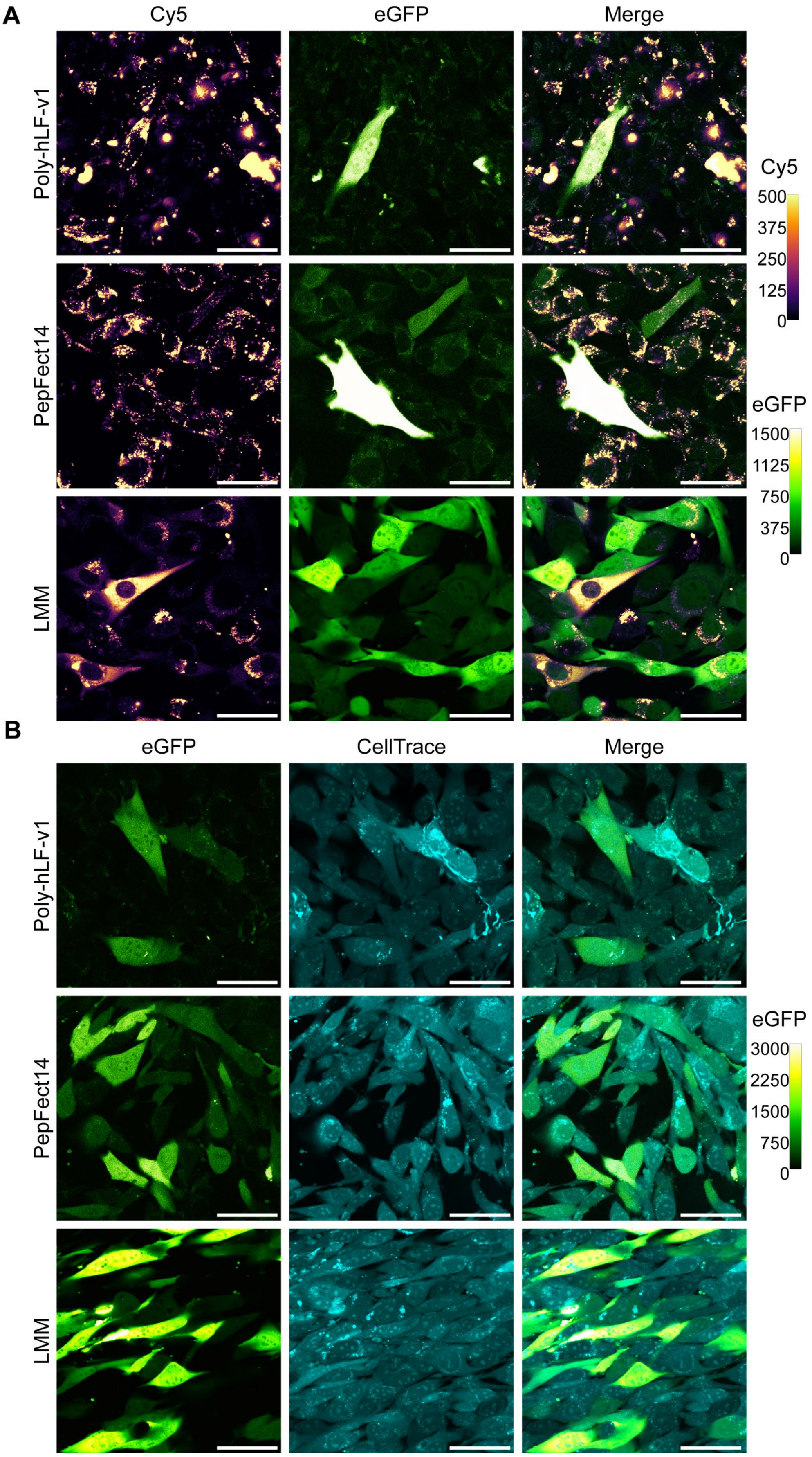
A
Cy5
eGFP
Merge
Poly-hLF-v1
PepFect14
LMM
Cy5
500
375
250
125
0
eGFP
1500
1125
750
375
0
B
eGFP
CellTrace
Merge
Poly-hLF-v1
PepFect14
LMM
eGFP
3000
2250
1500
750
0

Figure S7: For the lactoferrin variant, transfection is restricted to fewer cells than for PepFect14 and LMM. (**A**) Expression of Cy5-labeled eGFP mRNA 24 h post-transfection. (**B**) Expression of unlabeled eGFP mRNA 24 h post-transfection, cells were stained with 1 µM CellTrace Yellow. In both panels, the eGFP signal in LMM-transfected conditions were acquired with a 50-fold lower gain. Data are representative of three independent experiments. Scale bar represents 50 µm. Brightness and contrast of images were equally adjusted across conditions, per fluorophore, according to the look-up tables (right), where the values reflect pixel intensities.

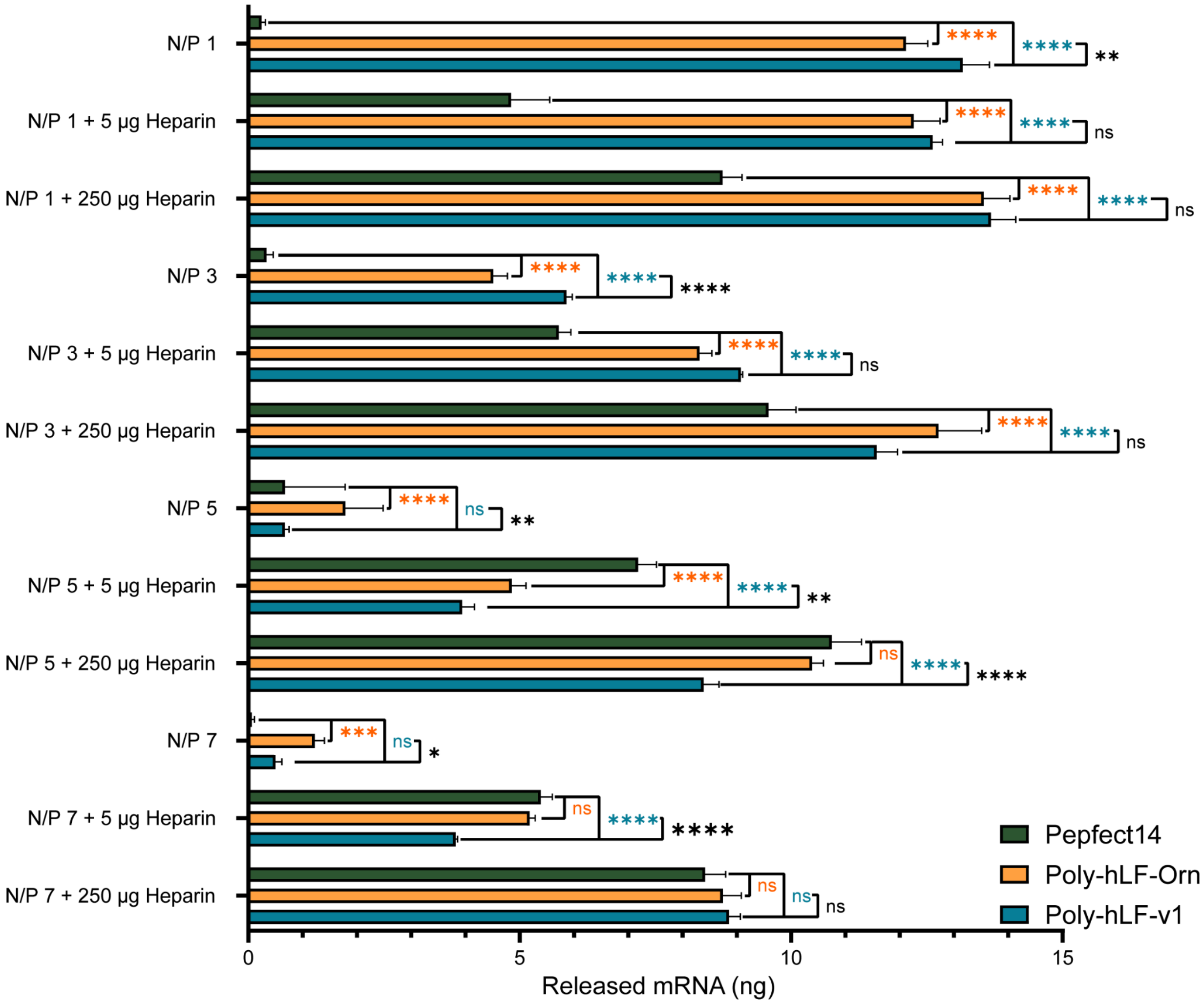

Figure S8: The complexation efficiency depends more strongly on the N/P ratio for lactoferrin variants than for PepFect14. For clarity, statistics are color-coded. Comparisons between PF14 and poly-hLF-Orn are depicted in orange, PF14 and Poly-hLF-v1 are depicted in blue, and poly-hLF-Orn and poly-hLF-v1 are depicted in black. Data are representative of 2 independent experiments, and data are represented as the mean + SD of four technical replicates. ns= non-significant, * $p \leq 0.05$, ** $p \leq 0.01$, *** $p \leq 0.001$, and **** $p \leq 0.0001$.

Supplemental Reference

(1) Mól, A. R.; Castro, M. S.; Fontes, W. NetWheels: A Web Application to Create High Quality Peptide Helical Wheel and Net Projections. bioRxiv 2018. https://doi.org/10.1101/416347.
